# Transcriptome profiling of Ewing sarcomas – treatment resistance pathways and IGF‐dependency

**DOI:** 10.1002/1878-0261.12655

**Published:** 2020-03-13

**Authors:** Yi Chen, Asle C. Hesla, Yingbo Lin, Mehran Ghaderi, Mingzhi Liu, Chen Yang, Yifan Zhang, Panagiotis Tsagkozis, Olle Larsson, Felix Haglund

**Affiliations:** ^1^ Department of Oncology‐Pathology Karolinska Institutet Stockholm Sweden; ^2^ Department of Molecular Medicine and Surgery Karolinska Institutet Stockholm Sweden; ^3^ Department of Orthopedic Surgery Karolinska University Hospital Solna Stockholm Sweden; ^4^ Department of Clinical Pathology and Cytology Karolinska University Hospital Solna Stockholm Sweden

**Keywords:** apoptosis, Ewing sarcoma, RNA‐seq, IGF2, transcriptome profiling, tumor progression

## Abstract

Ewing sarcomas (ESs) are aggressive sarcomas driven by *EWS* fusion genes. We sought to investigate whether whole‐transcriptome sequencing (RNA‐seq) could be used to detect patterns associated with chemotherapy response or tumor progression after first‐line treatment. Transcriptome sequencing (RNA‐seq) of 13 ES cases was performed. Among the differentially expressed pathways, we identified *IGF2* expression as a potential driver of chemotherapy response and progression. We investigated the effect of IGF2 on proliferation, radioresistance, apoptosis, and the transcriptome pattern in four ES cell lines and the effect of IGF2 expression in a validation series of 14 patients. Transcriptome analysis identified differentially expressed genes (adj. *P* < 0.005) and pathways associated with chemotherapy response (285 genes), short overall survival (662 genes), and progression after treatment (447 genes). Imprinting independent promoter P3‐mediated *IGF2* expression was identified in a subset of cases with aggressive clinical course. In ES cell lines, IGF2 induced proliferation, but promoted radioresistance only in CADO cells. High *IGF2* expression was also significantly associated with shorter overall survival in patients with ES. Transcriptome analysis of the clinical samples and the cell lines revealed an IGF‐dependent signature, potentially related to a stem cell‐like phenotype. Transcriptome analysis is a potentially powerful complementary tool to predict the clinical behavior of ES and may be utilized for clinical trial stratification strategies and personalized oncology. Certain gene signatures, for example, IGF‐related pathways, are coupled to biological functions that could be of clinical importance. Finally, our results indicate that IGF inhibition may be successful as a first‐line therapy in conjunction with conventional radiochemotherapy for a subset of patients.

## Graphical abstract

Transcriptome profiling may provide prognostic information in the incipient age of genetic‐driven personalized oncology. In this study, Chen *et al.* identified expression signatures associated with tumor progression and chemotherapy resistance in Ewing sarcomas, including *IGF2* expression which was associated with an aggressive clinical course. Transcriptome analysis could potentially become a complementary tool to predict the clinical behavior of rare tumors.

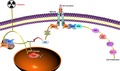

**Abbreviations**
ANOVA: analysis of variance
ATCC: America Type Culture Collection
B2M: beta‐2‐microglobulin
BSA: bovine serum albumin
CCK‐8: cell counting kit‐8
CDK2NA: cyclin‐dependent kinase inhibitor 2A
cDNA: complementary DNA
CSC: cancer stem cells
DAPI: 4′,6‐diamidino‐2‐phenylindole
EDTA: ethylenediaminetetraacetic acid
ERG: E26 transformation‐specific‐related gene
ESs: Ewing sarcomas
ETS: E26 transformation‐specific
EWS: Ewing sarcoma gene
FDR: false discover rate
FFPE: formalin‐fixed paraffin‐embedded
FITC: fluorescein isothiocyanate
FLI1: friend leukemia integration 1 transcription factor
GAPDH: glyceraldehyde 3‐phosphate dehydrogenase
Gy: Gray
HRP: horseradish peroxidase
IGF: insulin‐like growth factor
IGF‐1R: insulin‐like growth factor I
IGF2: insulin‐like growth factor II
IGFBP3: insulin‐like growth factor‐binding protein 3
IGV: Integrated Genome Viewer
ISG: Italian Sarcoma Group
ISO: International Organization for Standardization
LOI: loss of imprinting
MISO: mixture of isoforms
pAKT: phospho‐protein kinase B
PARP: poly(ADP‐ribose) polymerase
PCA: principal component analysis
pERK: phospho‐extracellular signal‐regulated kinases
PI: propidium iodide
QC: quality control
qRT–PCR: quantitative reverse transcription–polymerase chain reaction
RIPA: radioimmunoprecipitation assay
RNA‐seq: RNA sequencing
siRNA: small inhibitory RNA
SSG: Scandinavian Study Group
STAG2: stromal antigen 2
SWEDAC: Swedish Board for Technical Accreditation
SWI/SNF: SWItch/Sucrose Non‐Fermentable
TBST: tris‐buffered saline, 0.1% Tween 20
TKI: tyrosine kinase inhibitors
TP53: tumor protein 53

## 1 Introduction

Ewing sarcoma is the second most common primary bone malignancy in childhood and adolescence with an incidence of 2.9 per million/year in a population younger than 20 years of age. It is an aggressive tumor, genetically characterized by epigenetic remodeling induced by a fusion gene involving the *EWS* gene and an ETS transcription factor gene, most commonly (> 95%) the *FLI1* or *ERG* genes (Delattre *et al.*, 1992; Sorensen *et al.*, 1994). Genetic studies, including chromosome hybridization arrays as well as exome‐ and genome‐wide sequencing, have revealed a generally low mutational burden in ES (Brohl *et al.*, 2014; Crompton *et al.*, 2014; Shukla *et al.*, 2013). Recurrent secondary events include deletions of the *CDK2NA* gene and sporadic mutations in the *TP53* and *STAG2* genes, the two later having modest negative prognostic value (Brohl *et al.*, 2014; Huang *et al.*, 2005; Kovar *et al.*, 1993, 1997). Gene expression studies on clinical cases and cell lines have revealed prognostic gene transcription patterns associated with overall survival and transcriptional changes related to the fusion gene (France *et al.*, 2011; Ohali *et al.*, 2004; Volchenboum *et al.*, 2015).

Currently, most patients with ES receive neoadjuvant chemotherapy followed by surgery and/or radiotherapy. Standardized postoperative histopathological evaluation of tumor necrosis remains a strong prognostic marker and determines the continued treatment in, for example, the EWING 2008/2012 and the ISG/SSG III‐IV protocols (Biswas and Bakhshi, 2016; Elomaa *et al.*, 1999). Biomarkers of tumor chemotherapy response could optimize first‐line treatment protocols and follow‐up, allowing for early detection of patients who are not likely to respond to treatment.

In this study, we sought to investigate the transcriptome patterns of ES related to patient outcome, chemotherapy response, and tumor progression. To this end, we performed RNA sequencing of a single‐center cohort of a series of well‐characterized ES. Based on findings of increased *IGF2* gene expression in a few cases with very aggressive clinical course, we also investigated the functional role of IGF2 in ES cell lines.

## 2 Materials and methods

### 2.1 Patient samples and ethics

A total of 27 patients with ES were included in the study. RNA sequencing was performed on an exploratory cohort of 13 patients, and confirmatory experiments were performed on a validation cohort of 14 patients. All patients were treated according to the EWING 2008 or ISG/SSG IV protocols. For the exploratory cohort, cDNA was prepared from therapy‐naïve primary tumors and analyzed for the fusion gene as part of the clinical diagnostic workup (ISO‐validated and externally accredited method by SWEDAC) – all ESs with available cDNA at the time of the study were included. Ten of these cDNA samples were isolated from fine‐needle aspiration (FNA) material, and three samples were isolated from formalin‐fixed paraffin‐embedded (FFPE) specimens. The validation cohort used for immunohistochemistry and quantitative real‐time PCR (qRT–PCR) consisted of tumor samples from 14 patients and included primary tumors (*n* = 10, five of which were from chemotherapy‐treated patients) and metastatic lesions (*n* = 4).

The study and collection of patients’ samples were approved by the local ethics committee (The Regional Ethics Committee in Stockholm), reference number 2013 1979‐31. All patients had given oral and written consent prior to sample collection. Normal tissue controls were anonymized in accordance with the Swedish Biobank Law. The study methods were conformed to the ethical standards set by the Declaration of Helsinki.

### 2.2 Whole‐transcriptome shotgun sequencing

#### 2.2.1 Clinical samples

cDNA samples in the exploratory cohort were converted to double‐stranded cDNA (ds‐cDNA) using the SuperScript Double‐Stranded cDNA Synthesis Kit (Thermo Fisher Scientific, Waltham, MA, USA). Library generation, quality control, sequencing, and initial data processing were performed at the National Genomics Infrastructure, Science for Life Laboratory (SciLifeLab, Stockholm, Sweden), in Stockholm. Libraries were prepared using the Lucigen NxSeq AmpFREE Low Kit (Lucigen, Biosearch Technologies, Novato, CA, USA), quality‐controlled, and sequenced using the HiSeq 2500 (Illumina, San Diego, CA, USA) instrument with the HiSeq SBS Kit v.4 chemistry and 2x126‐bp paired‐end reading. Three samples in the exploratory cohort (which initially consisted of 16 cases) were excluded prior to sequencing based on insufficient library quality. The average sequence depth was 39.3 M reads (min 27.88–max 54.0 M reads). The Bcl‐to‐FastQ conversion was performed using the casava software (Illumina) suite. The quality scale was Sanger/phred33/Illumina 1.8+. Data processing followed the ‘NGI best practice pipeline’ which included raw quality control by FastQC, alignment to GRCh37 using tophat, alignment to QC using RSeqQC, and calculation of gene counts using HTSeq.

#### 2.2.2 Cell line sequencing

For functional validation experiments, RNA extracted from cell lines was analyzed at the National Genomics Infrastructure. Libraries were generated using TruSeq Kit and sequenced on a NovaSeq platform. Three samples were excluded prior to sequencing after QC control. The average read depth was 45.7 M reads (min 37.5–max 55.7 M reads). Data processing followed the ‘NGI best practice pipeline’ (which was updated between the two sequencing runs) including FastQC for raw quality control, Trim Galore! for adapter and quality trimming, STAR for alignment, RSeqQC for alignment QC, and featureCounts for calculation of gene counts.

### 2.3 Bioinformatic analysis

Differential gene expression analysis was performed using the deseq2 package (version 1.18.1) in rstudio (r version 3.4.1) with an adjusted *P*‐value (false discovery rate) cutoff of < 0.01 or < 0.005 as described in the Results section. The higher cutoff was used when no significant genes were found at the low cutoff. Comparative factors included fusion gene variant (*EWS‐Fli1* vs. *EWS‐ERG*), tumor localization (central vs. extremity), patient gender, age (< 20 vs. ≥ 20 years old), and survival at follow‐up. Response to chemotherapy was categorized as good or poor responders using the same histological criteria as the EWING 2008 and ISG/SSG IV protocols (<10% viable tumor cells; corresponding to Salzer‐Kuntschik grade 1‐grade 3). One patient was classified as a good responder based on complete radiological response.

First‐line treatment failure was defined as disease progression during or after the first line of treatment (including surgery, neoadjuvant chemotherapy, and radiotherapy). When comparing the transcriptome and clinical variables, we noted that one case frequently clustered together with tumors with more aggressive phenotype (poor chemotherapy response, first‐line treatment failure, and short overall survival). This small (5.5 cm) tumor presented in the extremity of a young boy. No neoadjuvant chemotherapy had been given because the diagnosis was established after complete tumor excision. We excluded this case from the first‐line treatment failure differential gene expression analysis (the case was still included in the PLA plots and heat maps for comparison).

Differentially expressed genes were compared to the Panther database (version 13.1) (Mi and Thomas, 2009; The Gene Ontology, 2017) for statistical overrepresentation using Fisher’s exact test and FDR correction for multiple testing. Overall differences between samples were depicted using principal component analysis (PCA) plots (deseq2 package). Statistically overrepresented pathways or biological processes were depicted using the pheatmap package (version 1.0.10). Furthermore, individual genes were manually plotted in spss (IBM Corp. Released 2016; IBM SPSS Statistics for Windows, Version 24.0; IBM Corp., Armonk, NY, USA).

Quantitative Sashimi plots were generated to visualize the promoter and isoform expression of *IGF2*. Reads were aligned to GRCh37 using star version 2.7.0 and quantified using a mixture‐of‐isoforms (MISO) model (Katz *et al.*, 2010, 2015) and visualized in the Integrated Genome Viewer (igv).

### 2.4 Cell cultures and reagents

A visual representation of the cell line experimental condition is shown in Fig. S1. Five Ewing sarcoma cell lines (RD‐ES, SK‐ES‐1, A673, SK‐N‐MC, and CADO) were maintained in the humidified atmosphere at 37 °C with 5% CO_2_ in RPMI 1640, McCoy’s 5A, DMEM, IMDM, and DMEM/F‐12 medium, respectively, with 10% fetal bovine serum. RD‐ES, SK‐ES‐1, and A673 were obtained from America Type Culture Collection (ATCC, Manassas, VA, USA). SK‐N‐MC and CADO were kindly provided by L. Girnita (Karolinska Institutet, Stockholm, Sweden). They were confirmed mycoplasma‐free using the Mycoalert™ Mycoplasma Detection Kit (Lonza, Basel, Switzerland). The characteristics of the five ES cell lines are summarized in Table 1 (including previously published data from Crompton *et al.*, 2014; Tirode *et al.*, 2014). The expression of *EWS/FLI* fusion gene was confirmed by RT–PCR in all cell lines. Recombinant human insulin‐like growth factor II (IGF2) was purchased from Sigma‐Aldrich (St Louis, MO, USA; Cat #I2526).

**Table 1:** Ewing sarcoma cell lines including donor and genetic information. NOS, not otherwise specified; WT, wild‐type; *: nonsense mutation (amino acid level).

ES cell line	Fusion gene	Donor age (years)	Donor gender	Site of tumor location	Cell line origin	Molecular characteristics^a^		
						*STAG2*	*CDKN2A*	*TP53*
RD‐ES	*EWS/FLI*	19	Male	Humerus	Primary ES	WT	WT	p.R273C
SK‐ES‐1	*EWS/FLI*	18	Male	Bone	ES‐NOS	p.Q735*	WT	C176F
A673	*EWS/FLI*	15	Female	Muscle	ES‐NOS	WT	del(1a,1b,2,3)	p.A119fs
SK‐N‐MC	*EWS/FLI*	14	Female	Supra‐orbital region	Askin's tumor (Ewing family of tumors)	p.M1_R546Del	WT	p.M1_T125Del
CADO	*EWS/FLI*	19	Female	Pleural effusion	Metastatic ES	WT	Homozygous deletion	WT

^a^ Tirode *et al. *(2014), Crompton *et al. *(2014).

### 2.5 Plasmids and transfections

Retroviruses that host the *CDKN2A* gene were packaged in Platinum‐A cells (Cell Biolabs, San Diego, CA, USA; Cat #RV‐102) that were transfected using pQCXIH‐CDKN2A from Addgene (Watertown, MA, USA; Cat # 37104). Supernatants of Platinum‐A cell cultures were collected, at 48, 72, and 96 h post‐transfection, filtered through 0.45‐µm polysulfonic filters, supplemented with 8 µg·mL^−1^ polybrene, and added to CADO cells for virus infection. The infected CADO cells were screened for stable *CDKN2A* integration by 500 ng·mL^−1^ hygromycin. After expedition, the expression of the *CDKN2A* gene products was confirmed by immunoblotting for p16.

For *STAG2* knockdown, CADO cells were transfected by Dharmacon™ ON‐TARGETplus pooled Human *STAG2* siRNA (Horizon, Cambridge, UK; Cat # L‐021351‐00‐0005) and DharmaFECT™ 1 Transfection Reagent (Horizon) according to the manufacturer’s instructions. STAG2 knockdown was confirmed by immunoblotting.

### 2.6 Irradiation of cells

Cells were seeded in 100‐mm dishes or 96‐well plates and cultured to 70–90% confluency before being serum‐starved for 24 h. After that, cells were irradiated in X‐RAD 225XL (North Branford, CT, USA) at doses of 6 or 25 Gy. Cells were put back to normal culture conditions after irradiation and were subsequently harvested at the indicated time points.

### 2.7 Cell proliferation assay

Ewing sarcoma cells were plated in 96‐well plates at a density of 5 × 10^3^ cells per well in serum‐free medium. The IGF2 (100 ng·mL^−1^) treatment was conducted 24 h after seeding and thereafter every 24 h with nontreated cells serving as control. The Cell Counting Kit‐8 (CCK‐8; Sigma‐Aldrich; Cat # 96992) was utilized to assess the proliferation of the cells at 0, 24, 48, 72, and 96 h after IGF2 treatment according to the manufacturer’s instructions. To test whether IGF2 could protect ES cells from irradiation, cells were exposed to X‐rays 4 h after the first dose of IGF2 and proliferation was monitored at 0, 24, 48, 72, and 96 h.

### 2.8 Flow cytometry analyses

Cells cultured in 100‐mm plates at 70–90% confluency were serum‐starved for 24 h prior to IGF2 stimulation (100 ng·mL^−1^). X‐ray exposure was conducted 4 h after the IGF2 treatment, and apoptosis was analyzed 24 h later employing the FITC Annexin V Apoptosis Detection Kit I (BD Biosciences, San Jose, CA, USA; Cat # 556547). Briefly, the harvested cells were washed twice with cold PBS and resuspended in binding buffer at a concentration of 1 × 10^6^ cells·mL^−1^. Then, 1 × 10^5^ cells from each sample were transferred into a 15‐mL Falcon tube. Next, 5 μL FITC Annexin V and 5 μL propidium iodide (PI) were added and incubated for 15 min at room temperature in the dark. After incubation, 400 μL of binding buffer was added to each tube and the samples were analyzed with Novocyte flow cytometry (Novocyte; ACEA Bio, San Diego, CA, USA). For the cell cycle analysis, the harvested cells were gently washed twice in PBS and then fixed in cold 70% ethanol for 30 min at 4 °C. After fixation, cells were washed twice in PBS and treated with 50 μL of a 100 μg·mL^−1^ ribonuclease in Falcon tubes. Next, 200 μL PI was added to each tube, and cell cycle analysis was conducted using Novocyte flow cytometry.

### 2.9 Immunofluorescence of PARP

Cells grown on cover slides were fixed with 4% paraformaldehyde in PBS for 15 min and permeabilized in 0.2% Triton‐X (Sigma) in PBS for 10 min at room temperature. After blocking in blocking buffer [5% normal goat serum (Thermo Fisher), 5% BSA (Thermo Fisher), and 0.2% Triton‐X 100 (Sigma) in PBS], the slides were incubated overnight at 4 °C in 1 : 400 anti‐cleaved PARP (Cell Signaling, Danvers, MA, USA; Cat # 5625) and diluted in antibody dilution buffer (1% goat serum and 0.2% Triton‐X 100 in PBS). Slides were then washed three times and incubated in secondary antibody anti‐rabbit Alexa Fluor 488 (Invitrogen, St Louis, MO, USA) at 1 : 100 dilution for one hour. After washing thrice, the slides were mounted in VECTASHIELD^®^ Antifade Mounting Medium with DAPI as counterstaining (Vector Laboratories, Burlingame, CA USA; Cat # H‐1200). The images were acquired using Zeiss Axio Imager M2 Microscope (Oberkochen, Germany) with consistent time and imaging processes. Ten random areas were chosen for statistical analysis.

### 2.10 Western blot

Total cell proteins were extracted using modified RIPA buffer [150 mm NaCl, 1% IGEPAL CA‐630, 0.025% sodium deoxycholate (Sigma), 50 mm Tris/HCl, 1 mm EDTA, 1 mm NaF, and X1 protease and phosphatase inhibitors (Thermo Fisher, Cat # 1861282)]. The cytoplasmic and nuclear proteins were fractionated using the Qproteome Cell Compartment Kit (Qiagen, Düsseldorf, Germany). The protein concentration was quantified by BCA Protein Assay Kit (Thermo Fisher; Cat # A33310). A total of 25 μg of protein from each sample was separated on NuPAGE™ SDS/PAGE (Thermo Fisher; Cat # NP0322BOX or Cat # NP0342BOX) and transferred to nitrocellulose membranes using electrophoresis. After blocking with 5% bovine serum albumin (BSA) in 1× TBST for 1 h, the membranes were incubated overnight at 4 °C with primary antibodies in appropriate dilutions. The primary antibodies recognizing IGF‐1R (#3027L; 1 : 800), pAKT (Thr308, #9275L; 1 : 800), pERK (Thr202/Tyr204, #9101L; 1 : 1000), total AKT(#9272S; 1 : 1000**)**, total ERK (#4696S; 1 : 1000), PARP (#9542; 1 : 800), cleaved PARP (#5625; 1 : 800), Caspase 3 (#9665; 1 : 800), cleaved Caspase 3 (#9664; 1 : 800), Caspase 9 (#9508; 1 : 800), cleaved Caspase 9 (#52873; 1 : 800), Caspase 7 (#12827; 1 : 800), cleaved Caspase 7 (#8438; 1 : 800), p16 (#4824S; 1 : 800), produced by Cell Signaling Technology, and Histone 3 (#sc‐10809; 1 : 1000), and GAPDH (#sc‐25778, 1 : 2000) provided by Santa Cruz Biotechnology (Dallas, TX, USA) were used for immunoblots at the indicated concentrations. Secondary goat anti‐rabbit HRP (Invitrogen; Cat #65‐6120; 1 : 2000) or goat anti‐mouse HRP (Invitrogen; Cat #62‐6520; 1 : 2000) were incubated at room temperature for 1 h after three washes in TBST. After washing, target proteins were visualized by chemiluminescence using peroxide solution and luminol enhancer solutions (Thermo Fisher; Cat #1859701, Cat #1859698) on X‐ray film.

### 2.11 Immunohistochemistry

The validation cohort (14 cases) was investigated for STAG2 and IGF2 protein expression using immunohistochemistry. In short, 4‐µm slides of FFPE tumor tissue were deparaffinized, rehydrated, and treated with low pH (citrate‐based) heat‐induced antigen retrieval for 20 min. Slides were incubated with either anti‐STAG2 (Santa Cruz; clone J‐12 sc‐81852; 1 : 100) or anti‐IGF2 (Abcam, Cambridge, UK; ab9574; 1 : 100). Positive and negative controls (normal placenta and liver) were included in each experiment. All slides were scored by a clinical pathologist.

### 2.12 Quantitative real‐time polymerase chain reaction

Areas with high tumor cell purity (> 85%) were isolated from FFPE blocks using 1‐mm punch biopsies after histological confirmation. Total RNA was extracted from clinical samples and cell lines using the RNeasy FFPE Kit or the RNeasy Plus Mini Kit, respectively (both from Qiagen), and reverse‐transcripted to cDNA using High‐Capacity RNA‐to‐cDNA™ Kit (Thermo Fisher; Cat # 4368814).

qRT–PCR quantification of target genes’ expression was performed with TaqMan™ Gene Expression Master Mix (Thermo Fisher; Cat No. 4369016) according to the manufacturer’s protocol using StepOnePlus Real‐time PCR System (Applied Biosystems, Foster City, CA, USA). The relative gene expression levels were calculated with the $2^{-\Delta \Delta C_{t}}$ method, where the *B2M* gene was used as internal control. To compare the gene expression between samples, an arbitrary relative expression level of 1 was set for one of the positive samples. Samples with a target gene *C*_t_ value > 35 and *B2M* *C*_t_ value < 30 (internal cDNA control) were classified as expression‐negative.

### 2.13 Statistics

We used Spearman’s ranked correlation when comparing continuous values. For comparing categorical variables in the clinical samples, the Mann–Whitney *U* or Kruskal–Wallis tests were used. Student’s *t*‐tests or ANOVA was used to analyze continuous variables in the cell line experiments. Overall survival was determined by the Kaplan–Meier analyses, and the log‐rank test was employed to determine statistical differences between groups. Cox regression was used for relative risk prediction. For overlap with previously published gene sets, we used exact hypergeometric probability with normal approximation. The statistics used to analyze the RNA‐seq experiments are described under the Bioinformatics section above. *P*‐values < 0.05 were considered statistically significant. False discover rate (FDR)/adjusted (adj.) *P*‐values were reported for multiple testing.

## 3 Results

### 3.1 Patient and tumor characteristics

The transcriptomes of 13 therapy‐naïve Ewing sarcomas were compared to identify patterns and mechanisms associated with different clinical and tumor properties. As described in the Materials and methods section, we used the clinical parameters response to chemotherapy (4 patients with good response vs. 9 patients with poor response, defined as viable tumor after chemotherapy (according to EWING 2008 or ISG/SSG IV criteria), metastasis at diagnosis or progression during therapy), first‐line therapy response (4 with tumor progression vs. 9 without), and overall survival (11 alive vs. 2 dead) to correlate with the transcriptome data. A summary of the clinical characteristics from the exploratory and validation cohorts is available in Table 2.

**Table 2:** Summary of clinical and tumor data from the discovery and validation cohorts. AWoD, alive without disease; AWD, alive with disease; DoD, dead of disease

Clinical characteristics	RNA‐seq cohort (*n* = 13)	Validation cohort (*n* = 14)
Analyzed material		
Primary tumor	13	10
Metastasis	0	4
Follow‐up		
Neoadjuvant chemotherapy	10	13
Histology		
Good responder	3	6
Poor responder	3	5
Radiology		
Complete response	1	1
‘Progression/nonresponder’	3	1
Metastasis at diagnosis	3	4
Progression after treatment	4	7
Classification (EWING 2008 and ISG/SSG IV)		
Good responder	4	7
Poor responder	9	7
Follow‐up (months)		
AWoD	8	4
AWD	2	3
DoD	2	6
Lost to follow‐up	1	1
Tumor characteristics		
Age at diagnosis (years)		
Median (min–max)	19 (2–33)	19 (5–48)
Sex		
Male	10	12
Female	3	2
Tumor characteristics		
Fusion gene		
EWS‐Fli1	11	7
EWS‐ERG	2	2
Other/ not specified	0	5
Primary tumor size (cm)		
Median (min–max)	8 (4–20)	7.5 (4.5–20)
Location		
Appendicular skeleton	11	9
Axial skeleton	2	2
Soft tissue	0	3

### 3.2 RNA‐seq revealed gene expression patterns associated with chemotherapy response and tumor progression

**Fig. 1:**
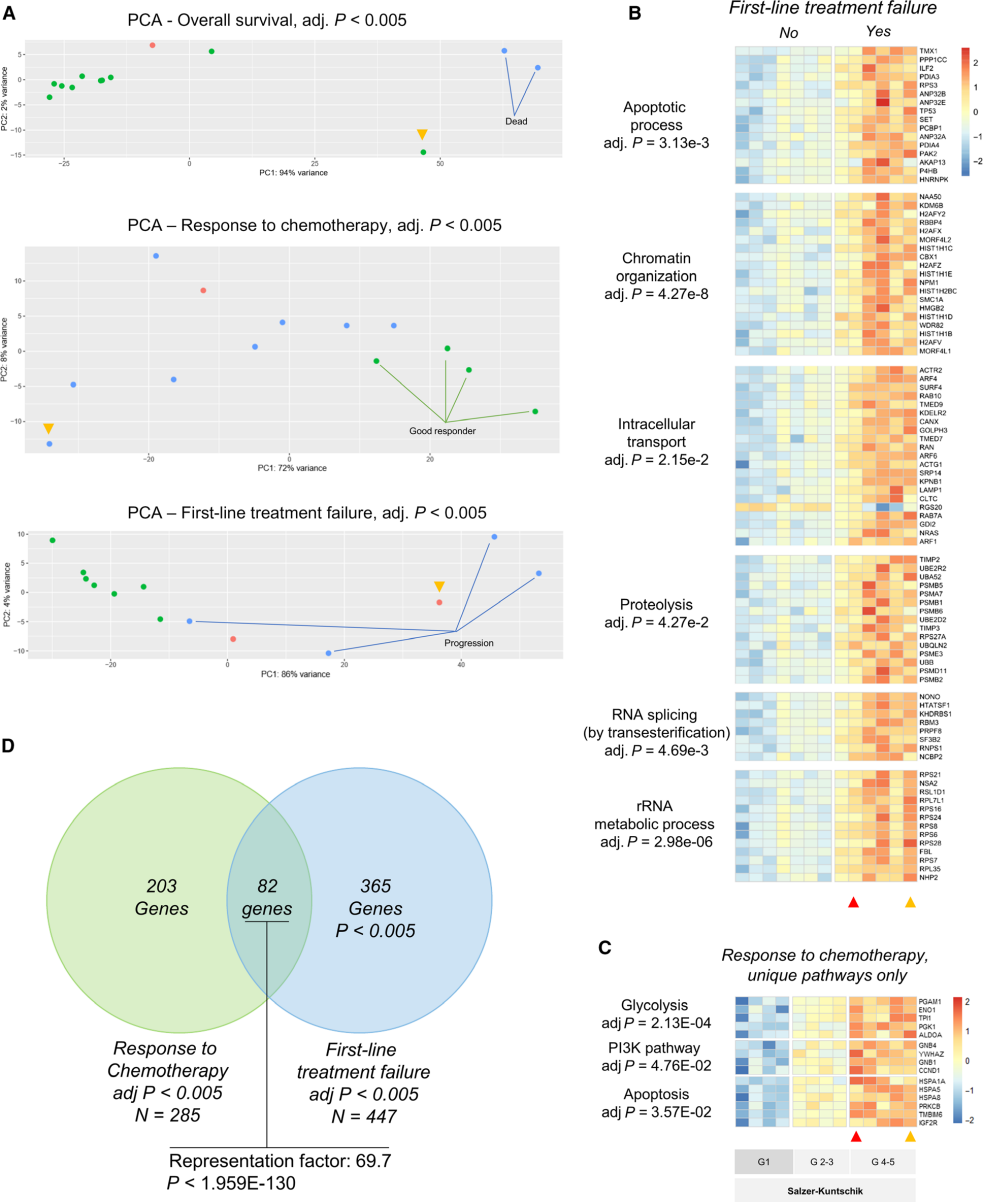
Differentially expressed genes and pathways in Ewing sarcomas. (A) Principal component analysis (PCA) depicting differentially expressed (DE) genes at adjusted *P* < 0.005. In the top plot (overall survival), green dots indicate living patients and blue dots dead patients at follow‐up. In the middle plot (response to chemotherapy), green dots indicate good response to chemotherapy (> 90% necrosis or complete radiological response) and blue dots indicate poor responders. In the bottom plot (first‐line treatment failure), blue dots indicate progression after therapy. In all PCAs, the red dots were not included (lost to follow‐up). The yellow arrow indicates the patient who was excluded from (first‐line treatment failure) analysis as described in the Materials and methods section. The heat maps depict expression levels of DE genes in six pathways linked to first‐line treatment failure (B) and three pathways only associated with response to chemotherapy (C). Tumor chemotherapy response was graded according to Salzer‐Kuntschik (G1–G5) as indicated. A Venn diagram (D) shows the overlap of genes involved in response to chemotherapy and first‐line treatment failure.

Principal component analysis (PCA) of differentially expressed genes at a significance level of *P* < 0.005 separated the tumors based on overall survival at follow‐up (662 genes, blue dots indicating dead patients), chemotherapy response (285 genes, green dots indicating good response), and first‐line therapy response (447 genes, blue dots indicating tumor progression) as shown in Fig. 1A and Tables [Link], [Link], [Link]. No separation of the cases was visible on the PCA plots (at *P* < 0.01) for patient age (0 genes), gender (19 genes), tumor localization (0 genes), or fusion gene subtype (32 genes) (data not shown). This suggests that (a) the transcriptome can explain, or at least visualize, the clinical behavior of ES, and (b) the patients’ underlying characteristics have limited influence on the ES transcriptome.

#### 3.2.1 Transcriptome characteristics of Ewing sarcomas with short patient survival

A PCA plot of differentially expressed genes showed that two patients who quickly died had large differences in the transcriptome (634 downregulated and 28 upregulated genes at adj. *P* < 0.005; Table S1) as shown in Fig. 1A (top plot, blue dots indicating dead patients). Overrepresentation test showed significant downregulation of genes coupled to anion transport (adj. *P* = 0.01), cell adhesion (adj. *P* = 0.001), cellular component morphogenesis (adj. *P* = 0.047), nucleotide metabolic process (adj. *P* = 0.015), and neuron synaptic transmission (adj. *P* = 5.0e‐9) as shown in Fig. S2. While the upregulated genes were fewer, several were highly upregulated (> 10‐fold) including the ligands *urotensin 2, cholecystokinin, insulin‐like growth factor 2,* and *periostin;* transcription factors *SOX9*, *BCL2,* and *MAFB;* and extracellular matrix components including *decorin* as well as *collagen types III* and *V.* While most downregulated pathways have known links to tumor development or progression, it is likely that the wide transcriptome downregulation is a reflection of a loss of dedifferentiation as reported by others (Danielsson *et al.*, 2013).

#### 3.2.2 Genes coupled to protein turnover and chromatin organization are significantly associated with first‐line treatment failure

In the initial analysis, we found many genes that were significantly associated with progression after chemotherapy (585 genes at adj. *P* < 0.01, Table S2). To identify the most significant genes, we decreased the *P*‐value cutoff, and at adj. *P* < 0.005, a total of 447 differentially expressed genes were identified (348 upregulated and 99 downregulated), as depicted in Fig. 1A, lower PCA plot. Several pathways were significantly overrepresented in cases with first‐line treatment failure: The heat map in Fig. 1B depicts the gene expression levels in significantly overrepresented pathways, which included upregulation of apoptotic process (adj. *P* = 3.13e‐3), chromatin organization (adj. *P* = 4.27e‐8), intracellular protein transport (adj. *P* = 2.15e‐2), proteolysis (adj. *P* = 4.27e‐2), RNA splicing by transesterification (adj. *P* = 4.69e‐3), and rRNA metabolic process (adj. *P* = 2.98e‐06). Interestingly, a majority of these genes were related to protein turnover functions, including ribosomal efficacy (rRNA metabolism, mRNA splicing), proteolysis, and intracellular protein transport. Also, several histones and histone acetyltransferases were upregulated which could result in dramatic epigenetic effects on chromatin organization.

#### 3.2.3 Genes encoding heat‐shock proteins, ribosomal proteins, or regulators of glycolysis are significantly associated with chemotherapy response

Fewer genes were significantly associated with chemotherapy response (410 genes at adj. *P* < 0.01, Table [Link], [Link]) as compared to first‐line treatment failure. At adj. *P* < 0.005, a total of 285 genes were upregulated in poor responders (Fig. 1A middle PCA plot). When the sequenced therapy‐naïve samples were arranged according to Euclidean hierarchal clustering, the cases distributed according to response grade (after completed neoadjuvant therapy) indicate that the response to chemotherapy could be predicted: Tumors with complete response (Salzer‐Kuntschik: grade 1, no viable cells according to histopathology) were separated from both poor responders (Salzer‐Kuntschik: grades 5–6) and good responders with incomplete response (Salzer‐Kuntschik: grades 2–3; 1–10% viable tumor cells). Statistical overrepresentation analysis revealed significant overrepresentation of genes related to the ribosome complex (adj. *P* = 2.55E‐03), heat‐shock proteins (adj. *P* = 9.22E‐04), mRNA splicing via spliceosome (adj. *P* = 2.23E‐03), CCKR signaling (adj. *P* = 1.82E‐02), or glycolysis (adj. *P* = 1.11E‐03) as visualized in the heat map (Fig. S3).

To identify which mechanisms were associated with chemotherapy response but not with tumor progression, we selected all genes that were exclusively found in the chemotherapy gene set (Fig. 1D). These genes (*n* = 203) were significantly related to glycolysis (adj *P* = 2.18e‐2), apoptosis (adj *P* = 3.57e‐2, which included many heat‐shock protein‐encoding genes), and the PI3K signaling pathways (adj *P* = 4.58e‐2).

### 3.3 Transcriptome relationship to EWS/FLI target genes

We compared our gene lists to a previously curated ‘core EWS/FLI1’ gene expression signature (503 upregulated and 293 downregulated genes) as proposed by Hancock and Lessnick (2008). This revealed a significant overlap with chemotherapy response [significantly increased overlap for the upregulated genes (37/410), *P* < 5.028e‐09], first‐line therapy failure [significantly increased overlap for the upregulated genes (28/513), *P* < 8.556e‐04], and patient survival [significantly increased overlap for upregulated genes (3/28), *P* = 0.045, and significantly decreased overlap for downregulated genes (3/634), *P* < 1.045e‐05] indicating that the level of the fusion protein activity (EWS/FLI1) could be important for the clinical tumor behavior (higher in nonresponders and perhaps less important in poorly differentiated cases).

### 3.4 Ewing sarcoma cancer stem cell markers

It has been proposed that cancer stem cells (CSCs) with higher glycolytic activity in ES may be responsible for chemotherapy resistance and disease progression (Dasgupta *et al.*, 2017; Fujiwara and Ozaki, 2016; Hotfilder *et al.*, 2018). We did indeed observe higher expression of previously suggested sarcosphere and side population markers in ES with first‐line therapy failure, including *STAT3*, *HIST1H2BC,* and *HIST1H1C*. Furthermore, as described below IGF2 stimulation of CADO cells increased three stem cell markers (*FOS, VMP1,* and *CYR61*), suggesting that IGF2 could induce a stem cell‐like phenotype.

### 3.5 Previously published ES gene expression data

We were unable to find a significant overlap with the 33 genes associated with poor patient outcome by gene expression microarray (Volchenboum *et al.*, 2015). Ohali *et al.* (2004) identified 818 genes that were significantly associated with poor patient outcome; however, these gene sets were not available for comparison. Other ES RNA‐seq publications did not include available clinical data (Brohl *et al.*, 2014; Crompton *et al.*, 2014).

### 3.6 IGF2 is expressed in a subset of ES with an aggressive clinical course

**Fig. 2:**
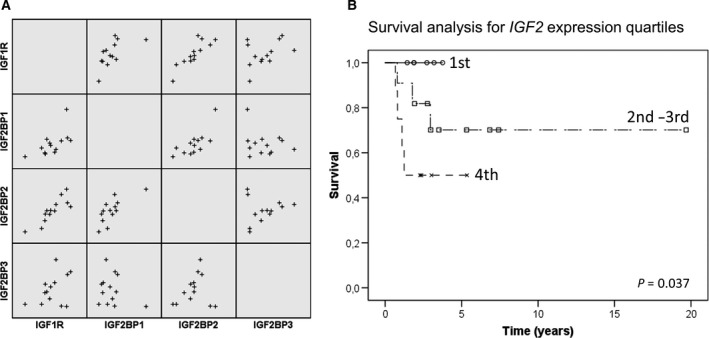
Co‐variation of IGF‐related genes and survival analysis based on *IGF2* expression in Ewing sarcomas: (A) Scatter plot showing expression correlation of four IGF‐associated genes in ES using RNA‐seq. There was a significant correlation (Pearson’s correlation, *P* < 0.05, *R* > 0.5) between many IGF‐related genes, including *IGF1R* and *IGF2R, IGF1BP3, IGF2BP1,* and *IGF2BP2*; or IGF2 and *IGF2BP1, IGF2BP2,* and *H19* (not all genes and correlates shown in the scatterplot). (B) The Kaplan–Meier curves comparing *IGF2* expression in ES using RNA‐seq (13 cases) and qRT–PCR (14 cases). Expression quartiles were calculated separately for the two methods. A log‐rank (Mantel–Cox) comparison showed significantly shorter survival with increased *IGF2* expression quartiles (*P* = 0.037).

When investigating individual transcripts in the gene sets, we observed higher levels of *IGF2* and lower levels of *IGFBP3* expression in cases with first‐line treatment failure or patient death at follow‐up. Upon further analysis, we observed a general co‐variation among IGF‐related genes across samples which could represent IGF dependence (exemplified as scatter plots in Fig. 2A). We hypothesized that cases with either high *IGF2* or *IGF1* expression could have a higher dependency on IGF signaling. Given the strong evidence of IGF‐1R signaling in ES, and its recent re‐actualization with combination therapies (Guenther *et al.*, 2019), we sought to further investigate the functional basis of IGF2 expression and its effects on ES cells.

We assessed the validation cohort of 14 ES patients for *IGF2* (IHC and qRT–PCR) and *H19* (qRT–PCR) expression and analyzed their potential relationship to patient survival. There was a significant association between *IGF2* gene and protein immunoreactivity (Fisher’s exact test, *IGF2* expression quartiles vs. IGF2 expression, *P* = 0.030) but not between IGF2 expression and *H19* expression (*P* = 0.580).

The relationship between IGF2 and patient survival was assessed by the Kaplan–Meier analysis. Gene expression data from the exploratory and validation cohorts were combined by looking at the expression quartiles of *IGF2*. There was a significant association (*P* = 0.037) between *IGF2* expression quartiles and overall survival as shown in Fig. 2B.

### 3.7 IGF2 induced proliferation in most ES cell lines

**Fig. 3:**
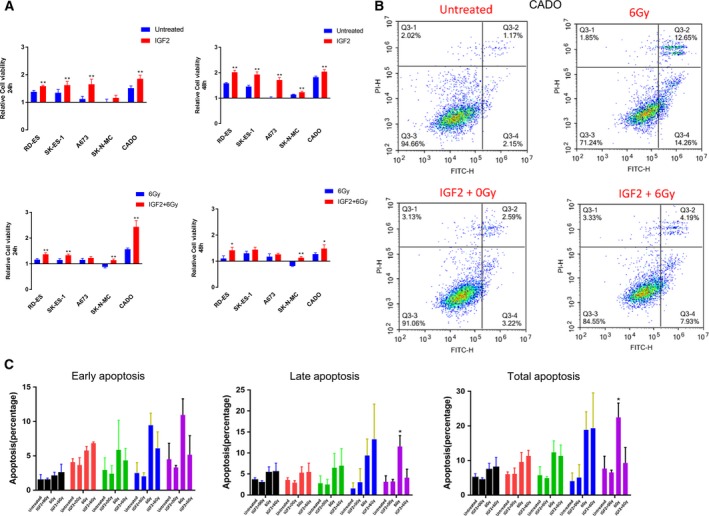
Effects of IGF2 on ES cell proliferation and cell apoptosis *in vitro*. ES cells were cultured in serum‐free medium for 24 h and stimulated with IGF2 (100 ng·mL^−1^) for 3 h prior to irradiation (6 Gy). (A) CCK‐8 assays were performed to detect the effects of irradiation and IGF2 stimulation on ES cell viability by 24 and 48 h. (B) Flow cytometry analysis showing the late (upper right) and early (lower right) apoptotic rates of CADO cells after irradiation and/or IGF2 stimulation, respectively. (C) Bar plots representing the percentages of early, late, and total apoptotic rates in RD‐ES (black), SK‐ES‐1 (red), A673 (green), SK‐N‐MC (blue), and CADO (purple) cells. Error bars represent the standard error of the mean from at least three independent experiments. *P < 0.05, **P < 0.01 (Student’s *t*‐tests).

Next, we investigated the effects of exogenous IGF2 stimulation on ES proliferation by CCK‐8 cell viability assay. As shown in Fig. 3A, IGF2 significantly increased cell proliferation in all cell lines but SK‐NM‐C by 24 h and the other cell lines by 48 h (*P* < 0.01).

### 3.8 IGF2‐induced radioresistance is dependent on AKT and ERK phosphorylation in ES cells

Since *IGF2* expression was associated with shorter patient survival and first‐line therapy failure, we investigated the effects of IGF2 stimulation on ES cell radiosensitivity. Cell viability was measured 24 and 48 h after irradiation (6 Gy) (Fig. 3A). By 24 h after irradiation, exogenous IGF2 increased cell viability in RD‐ES, SK‐ES‐1, SK‐N‐MC, and CADO cells (*P* < 0.01), but by 48 h the effects were significant only in RD‐ES, CADO, and SK‐N‐MC cells (*P* < 0.05). This effect was not mediated by cell cycle progression as flow cytometry showed no effects on G_2_/M transition (Fig. S4). In CADO cells, IGF2 significantly decreased total and late apoptosis after irradiation (Fig. 3B, *P* < 0.05), but this effect was absent or much weaker in the other cell lines (Fig. 3C, Fig. S5). Altogether, these data demonstrate that the radioprotective effects of IGF2 were limited to the CADO cell line.

**Fig. 4:**
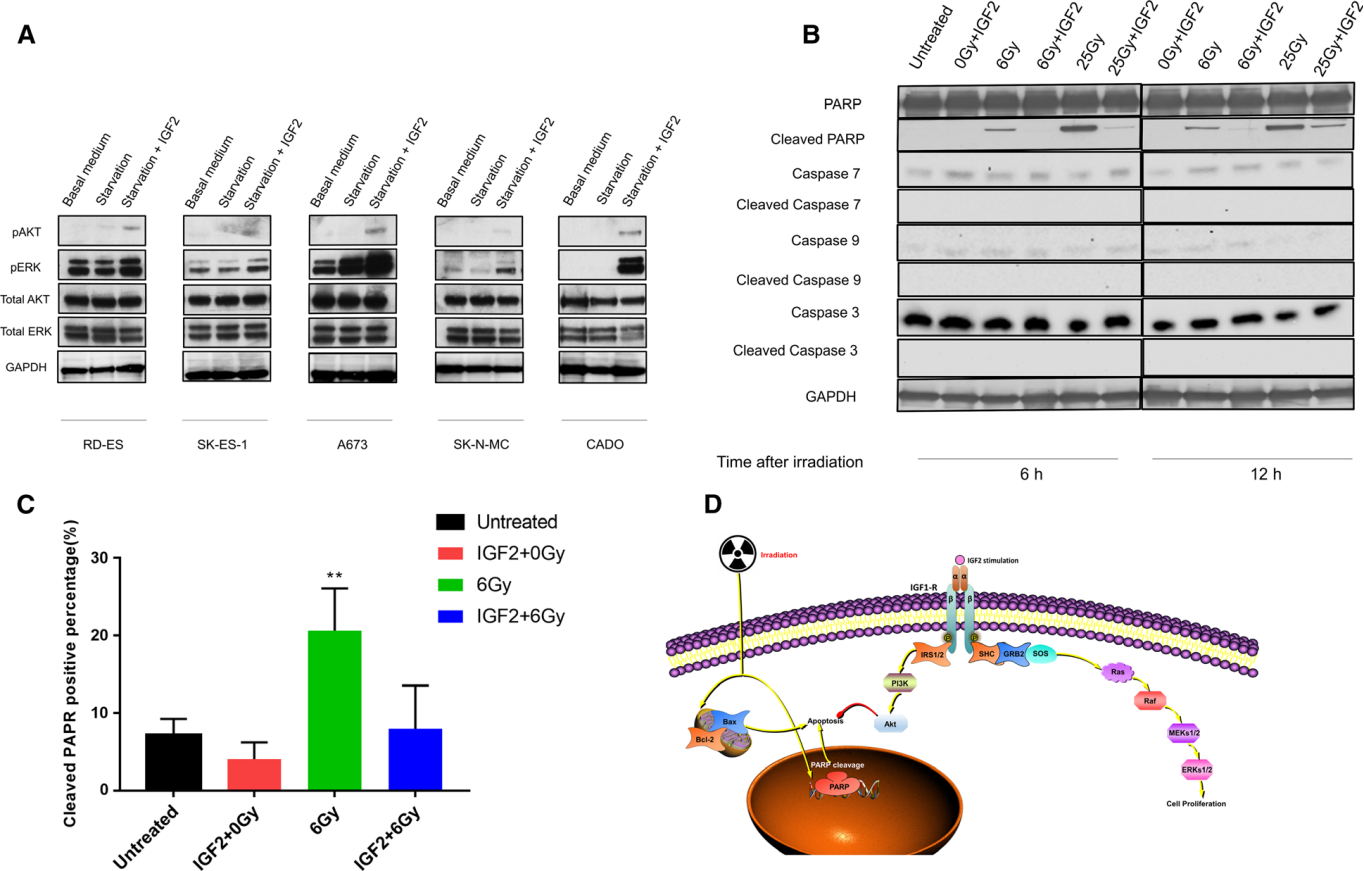
IGF2 contributes to the development of radioresistance in ES lines based on AKT and ERK phosphorylation. ES cells were cultured at serum‐free medium for 24 h, and then stimulated with IGF2 (100 ng·mL^−1^) for 0.5 h (A) or 3 h (B–D) prior to irradiation (6 Gy). Western blot analysis (A) showing IGF2/IGF‐1R signal transduction in ES cells, including pAKT, pERK, total AKT, and total ERK following IGF2 stimulation, and PARP and Caspase cleavage following irradiation (6 or 25 Gy) and IGF2 stimulation in CADO cell by 6 and 12 h. GAPDH was stained as a loading control. (C) Bar plots representing the proportion of cleaved PARP after irradiation and/or IGF2 stimulation as determined by an immunofluorescence assay. Error bars represent the standard error of the mean from at least ten random areas. *P < 0.05, **P < 0.01 (Student’s *t*‐test). (D) Schematic model of IGF2/IGF1R‐induced AKT and ERK signaling in Ewing sarcoma cells, including increase in proliferation and PARP‐mediated radioresistance and decreased apoptosis.

To investigate the mechanism underlying these radioprotective effects, we investigated the IGF2/IGF‐1R signaling in the ES cell lines. While external IGF2 triggered varying levels of AKT and ERK phosphorylation in the investigated cell lines, all cell lines but CADO had a baseline ERK phosphorylation (Fig. 4A). In CADO cells, AKT phosphorylation and ERK phosphorylation were distinctly increased upon IGF2 stimulation. The A673 cells were the least responsive to IGF2 in terms of proliferation and radioresistance, and this cell line also showed the highest baseline pERK level (Fig. 4A). These data suggest that the unresponsiveness to IGF2 in RD‐ES, SK‐ES‐1, SK‐N‐MC, and A673 cells is caused by constitutive activation of Ras signaling.

### 3.9 IGF2‐mediated signaling prevents PARP cleavage in the CADO Ewing sarcoma cells

To investigate the mechanism of action underlying IGF2‐induced radioresistance in CADO cells, we studied the levels of apoptosis‐related proteins (total and cleaved PARP, total and cleaved Caspases 3, 7, and 9) after irradiation treatment (6 and 25 Gy) at 6 and 12 h. As shown in Fig. 4B, the addition of IGF2 drastically decreased the levels of cleaved PARP at both time points. However, protein levels of cleaved Caspases 3, 7 and 9 remained unchanged. PARP cleavage was also examined using immunofluorescence, showing that IGF2 treatment of CADO cells inhibited cleavage of PARP after 6 Gy of irradiation (Fig. 4C, Fig. S6). These results suggest that irradiation triggers Caspase‐independent apoptosis in ES cells and that IGF2 is able to inhibit this signaling pathway.

### 3.10 Gene expression alteration in Ewing cell lines after IGF2 stimulation

**Fig. 5:**
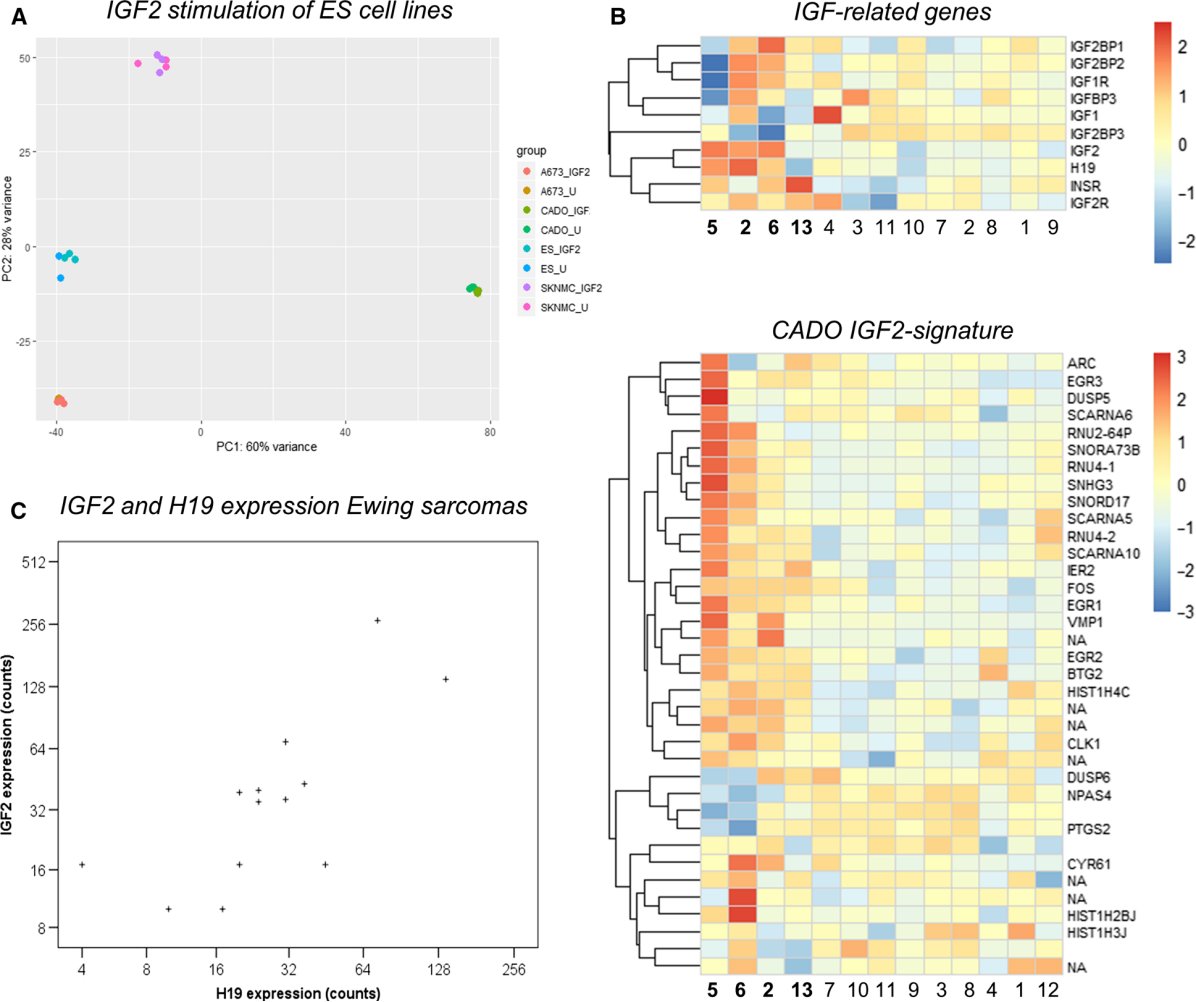
IGF2 induced changes in ES cell lines and IGF2 signatures in clinical ES. (A) Principal component analysis (PCA) plot of all genes in four ES cell lines with and without IGF2 stimulation. Dot colors indicate cell line (A673, CADO, ES‐1, or SKNMC) and IGF2 treatment condition (IGF2 = 100 ng·mL^−1^ of IGF2 treatment, U = untreated) for three replicates per condition as shown in the legend to the right. The clearest separation was seen in the CADO cells. (B) Heat maps showing expression levels of IGF‐related genes and the CADO IGF2 signature (differentially expressed after IGF2 stimulation) in Ewing sarcomas identified by RNA‐seq. Euclidean clustering of cases showed a similar separation of cases with bona fide higher IGF‐signaling dependency (cases 5, 6, 2, and 13). (C) Scatter plot showing a significant correlation (Pearson’s correlation; *P* = 0.010 and *r* = 0.683) between *IGF2* expression and *H19* gene expression in Ewing sarcomas by RNA‐seq.

To understand the different responses to IGF2 in ES cell lines, we stimulated ES cells for a short duration of time (30 min) and performed transcriptome sequencing (Fig. 5A). As expected, IGF2 induced different transcriptome responses in different ES cell lines. Based on the poor separation of replicates of SK‐NM‐C and RD‐ES in the PCA plot (Fig. S7), we concluded that their results would be unreliable. Differential expression analysis at adj. *P* < 0.01 identified 36 genes in CADO (Table S4), 84 genes in SK‐ES‐1 (Table S5), and 2 genes in A673 cells (Table S6). Indeed, CADO, SK‐ES‐1, and A673 showed similarities in gene response inducing *FOS*, *EGR1,* and *DUSP6* expression. We then plotted the CADO signature (36 DE genes) in a PCA plot which revealed a separation between treated and untreated cells in all cell lines (Fig. S8), suggesting that these genes are general responders to IGF2 treatment (albeit at nonsignificant levels for individual genes in the other cell lines).

Enrichment analysis of the CADO signature showed upregulation of ‘inactivation of MAPK activity’ (adj. *P = *1.97E‐2) consistent with a negative feedback loop, and downregulation of *‘*nucleosome assembly’ (adj. *P = *7.05E‐3). In terms of individual genes, the signature contained a stark increase in many immediate‐early genes encoding transcription factors (e.g., *FOS*, *EGR1‐3*, *IER2, BTG2, NPAS4,* and *ARC*) as well as upregulation of *PTGS2* (encoding COX2). Interestingly, we also observed a distinct downregulation of histone encoding genes (*HIST1H3J, HIST1H4C,* and *HIST1H2BJ*) and spliceosome‐associated small nuclear ribonucleoproteins (RNP) (sno/scaRNAs: *SNORD17, SNORA73B, SCARNA6, SCARNA10, SCARNA5*).

We also looked at the IGF receptors and IGF‐related genes in the ES cell lines. All cell lines had low *IGF2* expression, but CADO cells had high *IGF1* expression suggesting an existing autocrine loop. CADO also had the highest expression levels of *IGF1R*, while CADO and ES‐1 also had high *INSR* expression (data not shown). There was a clear similarity in the Euclidean clustering of clinical cases when comparing the CADO gene signature with IGF‐related genes (Fig. 5B). This strengthens the hypothesis that multiple genes determine the responsiveness and dependency of IGF‐related signaling in clinical cases of ES.

### 3.11 IGF2 expression is not caused by loss of imprinting, *STAG2* or *CDKN2A* gene inactivation

It has previously been reported that loss of imprinting at the *IGF2*/*H19* locus is not a determinant of *IGF2* expression in ES (Zhan *et al.*, 1995). In line with this conclusion, we found a strong linear relationship (Fig. 5C, Spearman’s ranked correlation, *P* = 0.003, *r* = 0.750) between *IGF2* expression and *H19* expression suggesting a regulatory mechanism independent of loss of imprinting (which presumes an inverted relationship). We mapped individual transcripts to the *IGF2* exons using MISO and visualized them in Sashimi plots (the two aggressive ES cases expressing *IGF2* are exemplified in Fig. S9). This shows that the *IGF2* promoter 3 (P3) is the dominating transcription initiation site (no transcripts were detected from P1). It has previously been shown that loss of imprinting (LOI)‐independent hypomethylation of P3 induces *IGF2* expression in osteosarcoma, and ovarian and prostate cancer (Kuffer *et al.*, 2018; Li *et al.*, 2009; Murphy *et al.*, 2006); however, several transcription factors have been known to regulate P3 methylation and activity, for example, Paxillin, STAT3, and PLAG1 (Akhtar *et al.*, 2012; Huang *et al.*, 2014; Lee *et al.*, 2016; Marasek *et al.*, 2015).

We investigated whether the *IGF2/H19* expression could be triggered by either *STAG2* mutations or *CDKN2A* deletion given the known role of these events in ES. Experimentally, this was investigated in CADO cells by siRNA knockdown of *STAG2* expression [CADO is *STAG2* wild‐type as described by Huang et al. Huang *et al.*, 2011 (Tirode *et al.*, 2014)] and by *CDKN2A* overexpression in CADO cells [which are known to harbor a homozygous *CDKN2A* deletion (Ottaviano *et al.*, 2010)]*.* Opposite to our hypothesis, *STAG2* knockdown resulted in decreased levels of *IGF2* and *H19* (Fig. S10). *CDKN2A* overexpression in CADO did not alter the *IGF2* gene expression but significantly increased *H19* levels (Fig. S10). Furthermore, loss of STAG2 immunohistochemistry in the clinical samples was not associated with higher IGF2 expression (Fig. S11). Collectively, these data suggest that *IGF2* expression is triggered by a combination of epigenetic and transcription factors rather than a single specific mutational event.

## 4 Discussion

In this paper, we investigated the transcriptomes of 13 ES cases. We identified gene signatures that were significantly associated with short patient survival, chemotherapy response, and progression after first‐line treatment. High expression of IGF‐related proteins and targets has previously been identified in a subset of ES with aggressive phenotype (van de Luijtgaarden *et al.*, 2013). In line with their observations, we identified a subset of especially aggressive ES with high expression of IGF2 and IGF target genes. *In vitro*, IGF2 stimulation increased proliferation in several ES cell lines, whereas significantly decreased apoptosis and increased radioresistance were exclusive to CADO cells. IGF2 stimulation of CADO cells generated an immediate‐early gene response pattern (Fig. 5B) which corresponded to expression levels of IGF‐related genes in clinical samples, possibly distinguishing a clinically significant IGF‐dependent gene expression signature. Alongside IGF‐related signaling, we identified several pathways that were significantly associated with chemotherapy resistance, tumor progression, and patient outcome as discussed below.

While all ESs are considered high‐grade sarcomas, the clinical outcome is still variable. Tumor DNA sequencing has shown that *STAG2* mutations (Brohl *et al.*, 2014; Crompton *et al.*, 2014) or chromoplexy‐associated fusion gene genesis (Anderson *et al.*, 2018) is associated with more aggressive clinical behavior. Also, recurrent tumors and metastatic lesions had an increased number of genetic aberrations (Brohl *et al.*, 2014). While transcriptome studies (RNA‐seq) of patient samples have been performed previously, no prognostic patterns have been defined.

In our cohort, there was a significant overlap between genes associated with tumor progression after first‐line therapy and chemotherapy response (Fig. 1D, *P* < 1.9E‐130). Tumor necrosis after chemotherapy is currently the best tool for determining the prognosis of ES patients (Biswas and Bakhshi, 2016; Elomaa *et al.*, 1999), but this may be improved by RNA‐seq analysis. Our results identified PI3K signaling, glycolysis, and heat‐shock protein genes to be associated with reduced chemotherapy response, but not for progression after therapy (Fig. 1B,C). While PI3K signaling can be targeted by tyrosine kinase inhibitors (TKI), these pharmacological regimes are frequently saved for second‐ or third‐line therapy. Similar to the conclusions drawn by van de Luijtgaarden et al., our results indicate that TKI inhibitors could be more effective in combination with first‐line chemotherapy.

It has been proposed that CSC with high glycolysis exists within ES (Dasgupta *et al.*, 2017; Fujiwara and Ozaki, 2016; Hotfilder *et al.*, 2018). We observed overlap between genes induced by EWS/FLI1, expression of genes linked to chromatin organization (e.g., histones), IGF2‐induced genes (in CADO cells), and chemotherapy/progression‐related genes in patient samples (partly shown in Fig. 5B, gene expression data extracted from Dasgupta *et al.*, 2017; Fujiwara and Ozaki, 2016; Hancock and Lessnick, 2008; Hotfilder *et al.*, 2018). These data indicate a relationship between ES cell metabolism, EWS/FLI1 activity (linked to mSWI/SNF), and resistant/aggressive tumor phenotype. If a subpopulation of ES‐CSC indeed exists, it should be detectable by single‐cell RNA‐seq which is a possible continuation of this study.

Another novel and potentially important finding is the observation that high expression of ribosomal subunits corresponded to both decreased chemotherapy response and progression after treatment, suggesting that the ribosomal biogenesis inhibitors (which are in early clinical development, e.g., CX‐5461, identifier NCT02719977) should be investigated in ES.

IGF signaling and IGF‐1R signaling have previously been extensively investigated in ES (summarized by Olmos *et al.*, 2011). While clinical trials showed durable responses in a subset (10–14%) of patients, usable biomarkers for IGF‐targeted therapy are yet to be established. Perhaps the most promising study has utilized post‐IGF‐1R inhibition FDG‐PET to measure tumor metabolic activity as a potential biomarker (van Maldegem *et al.*, 2016). A recent experimental study suggests that combination therapy with CDK4/6 and IGF‐1R inhibitors may be more successful to target ES, showing that more complex studies of ES IGF signaling and dependency are required to increase IGF‐targeted treatment efficacy (Akhtar *et al.*, 2012; Hyun *et al.*, 2016). While our transcriptome analysis is limited to pretreatment data and lacks the dynamics of an *ex vivo* model, the co‐expression of IGF‐related genes suggests that transcriptome analysis can be used to identify IGF dependency in ES, potentially acting as a treatment biomarker. Another important observation is that *IGF2* expression was accompanied by changes in other pathways (Fig. 1B,C) which could indicate that these tumors are more resilient as compared to other ESs. This could also indicate that tumors that initially respond to IGF inhibition also harbor the ability to change pathway dependencies.

Since *IGF2* expression was linear with *H19* expression and initiated by the LOI‐independent P3 promoter (Fig. 5C, Fig. S9), we were able to partly explain the mechanisms behind *IGF2* expression in ES. Unfortunately, we were unable to identify the specific mechanism underlying *IGF2* expression, including *CDKN2A* and *STAG2* inactivation.

## 5 Conclusions

In conclusion, we have identified multiple gene expression patterns linked to patient survival, tumor progression, and chemotherapy resistance in Ewing sarcoma. While the lack of large validation cohorts limits the generalizability of these gene signatures from a prognostic perspective, our results identify several oncogenic pathways which can be subject to further studies including IGF2‐autocrine loops, IGF dependency, ES stem cells, and the potential effects of ribosomal biogenesis inhibitors. Importantly, our results provide a strong rationale to continue the investigation of RNA sequencing in the context of individualized clinical treatment.

## Conflict of interest

The authors declare no conflict of interest.

## Author contributions

YC, YL, ML, and CY performed the experiments; YC, YL, MG, ML, and FH conducted the data analysis; ACH, YZ, PT, and FH provided the clinical samples and clinical data; YC, YL, OL, and FH planned the project; and YC and FH prepared the manuscript.

## Supporting information


**Fig. S1. **Flow chart depicting experimental conditions of cell line experiments.

**Fig. S2. **Heat map showing the differentially expressed genes in significantly overrepresented pathways associated with *overall survival (good = alive, poor = dead).*

**Fig. S3. **Heat map showing the differentially expressed genes in significantly overrepresented pathways associated with *response to chemotherapy*.

**Fig. S4. **Flow cytometry of CADO cells showed no significant changes in cell cycle progression (amount of cells in G2/M phase) after IGF2 stimulation with or without irradiation (6 Gy).

**Fig. S5.** Flow cytometry was performed to determine the apoptotic rates of A673, SK‐ES‐1, RD‐ES and SK‐N‐MC ES cell lines. Two or more experiments were performed in each cell line. Quantification of the flow cytometry experiments are visualized as bar plots in Figure 3C. Upper right square = late apoptotic cells; Lower right square = early apoptotic cells; Upper right and lower right squares = total apoptotic cells.

**Fig. S6. **Immunofluorescence assays were conducted to detect how the levels of cleaved PARP was related to irradiation (6 Gy) with or without IGF2 stimulation in CADO cells.

**Fig. S7. **Principal component analysis (PCA) plot showing all genes in IGF2‐treated and untreated cell lines. Dots indicate individual replicates (n = 2 ‐ 3) and color represents cell line type and treatment (IGF2 = 100ng/ml IGF2 treatment, U = untreated) as indicated by the legend to the right. Poor separation was seen for RD‐ES cells which were excluded from further analysis.

**Fig. S8. **Principal component analysis (PCA) plot of all cell lines for the gene signature (36 differentially expressed genes) related to IGF2 stimulation of CADO cells. The PCA shows a variable separation (for each individual cell line) of treated and untreated experimental replicates.

**Fig. S9. **Sashimi plot showing the mapping of RNA‐seq reads to the *IGF2* exons for a sample with *IGF2* expression. Similar to the other cases with *IGF2* expression on RNA‐seq, the mapping reveals how promoter P3 is the clearly dominant site for transcription initiation. Promoter P1 is not shown, but had a very low number of mapped reads for all samples. This suggests that the gene expression of *IGF2* is not mediated by loss of imprinting.

**Fig. S10. **A) qRT–PCR analysis of *IGF2* and *H19* expression in untransfected control and siSTAG2‐treated CADO cells. B) Western blotting of P16 in Hela (positive control), uninfected CADO and *CDKN2A* encoding retrovirus infected (pQCXIH‐*CDKN2A*) CADO cells. GAPDH was stained as a loading control. C) qRT–PCR analysis of *IGF2* and *H19* expression in uninfected CADO and *CDKN2A* encoding retrovirus infected (pQCXIH‐*CDKN2A*) CADO cells. *p <0.05, **p <0.01.

**Fig. S11. **Representative photomicrographs of immunohistochemical staining for STAG2 in Ewing sarcomas.

**Table S1. **Differentially expressed genes for patient survival in Ewing sarcoma patients at adj. *p* < 0.005.

**Table S2. **Differentially expressed genes for First‐line therapy failure in Ewing sarcoma patients at adj. *p* < 0.01.

**Table S3. **Differentially expressed genes for response to chemotherapy in Ewing sarcoma patients at adj. *p* < 0.01.

**Table S4. **Differentially expressed genes in CADO at adj. *p* < 0.01.

**Table S5. **Differentially expressed genes in SK‐ES‐1 at adj. *p* < 0.01.

**Table S6. **Differentially expressed genes in A673 at adj. *p* < 0.01.
