# Supplementary figures and images for: Transcriptome profiling of Ewing sarcomas – treatment resistance pathways and IGF‐dependency
Source: Mol Oncol. 2020 Mar 13;14(5):1101–17. doi: 10.1002/1878-0261.12655 (PMC7191197; doi:10.1002/1878-0261.12655)

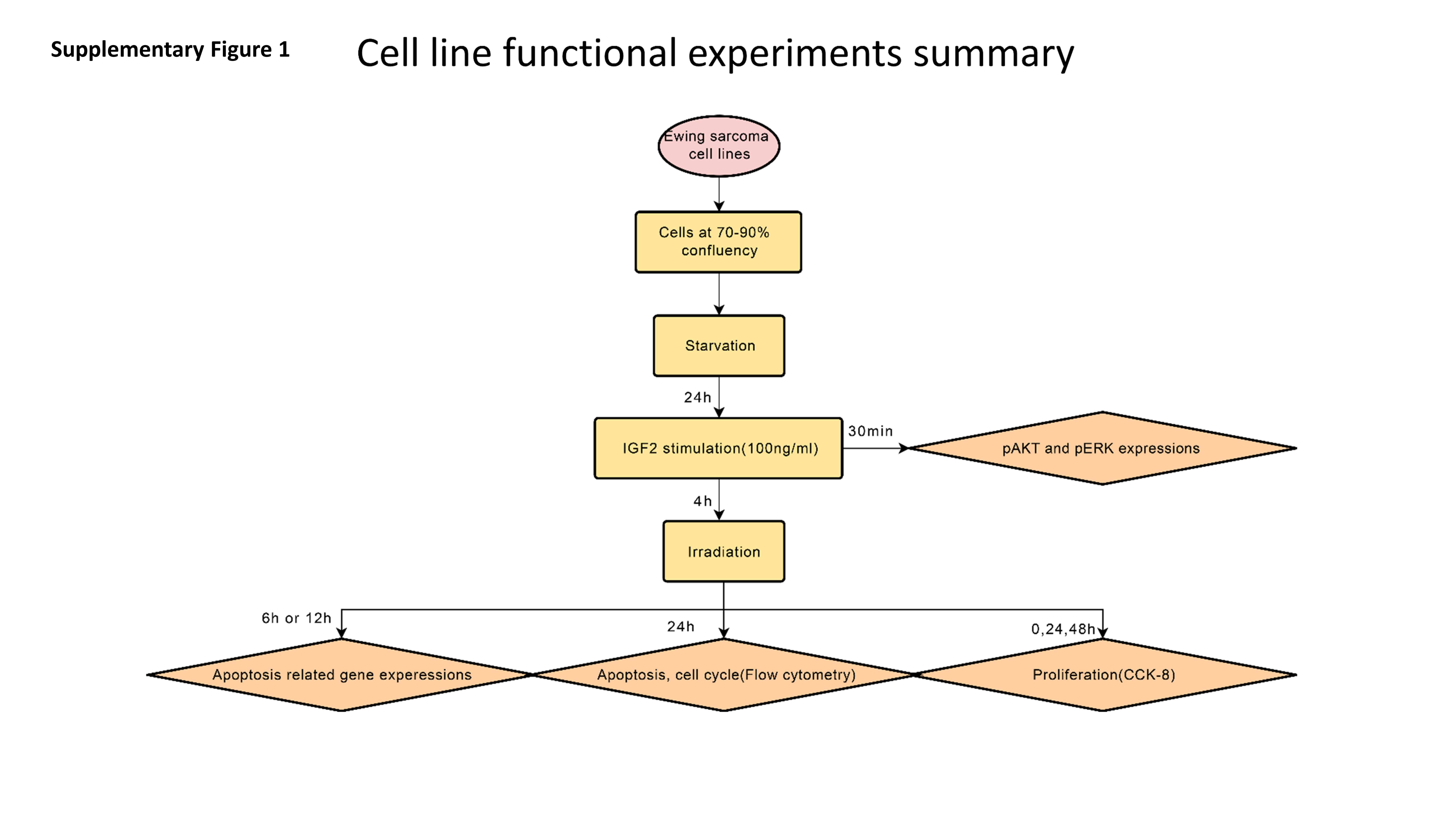

Supplement: Supplementary file 1 — Fig. S1. Flow chart depicting experimental conditions of cell line experiments. [file MOL2-14-1101-s001.tif]

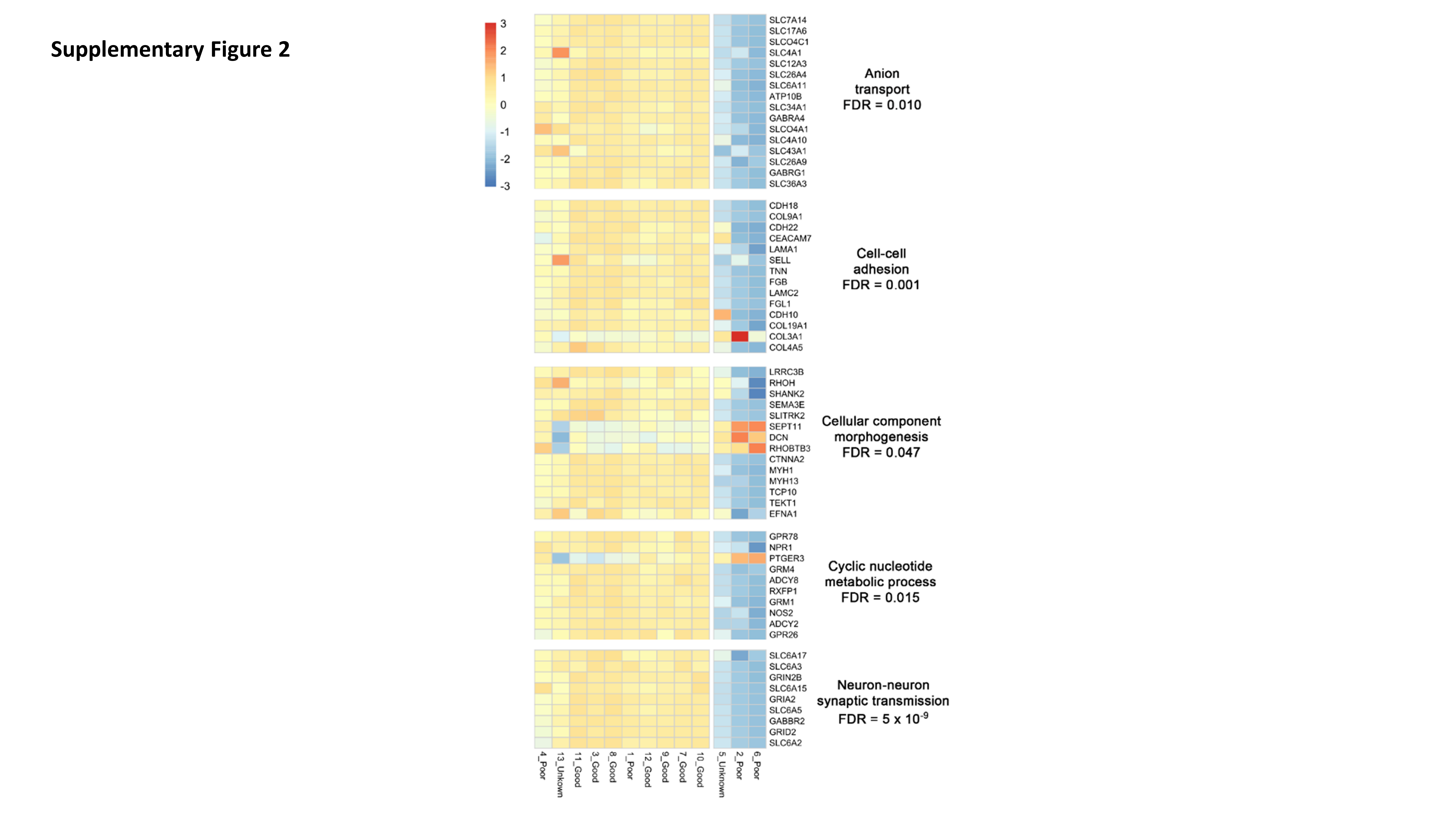

Supplement: Supplementary file 2 — Fig. S2. Heat map showing the differentially expressed genes in significantly overrepresented pathways associated with overall survival (good = alive, poor = dead). [file MOL2-14-1101-s002.tif]

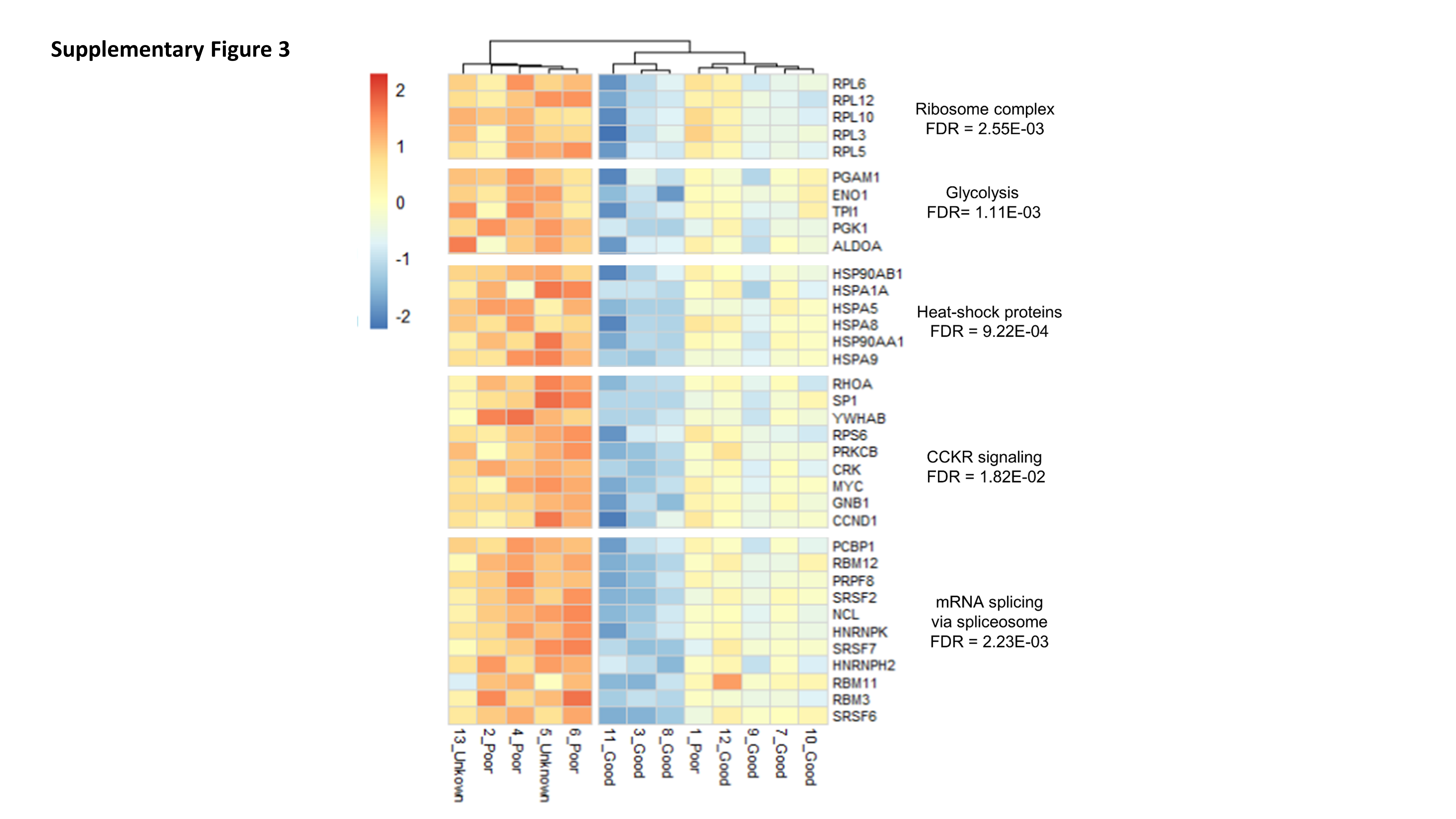

Supplement: Supplementary file 3 — Fig. S3. Heat map showing the differentially expressed genes in significantly overrepresented pathways associated with response to chemotherapy. [file MOL2-14-1101-s003.tif]

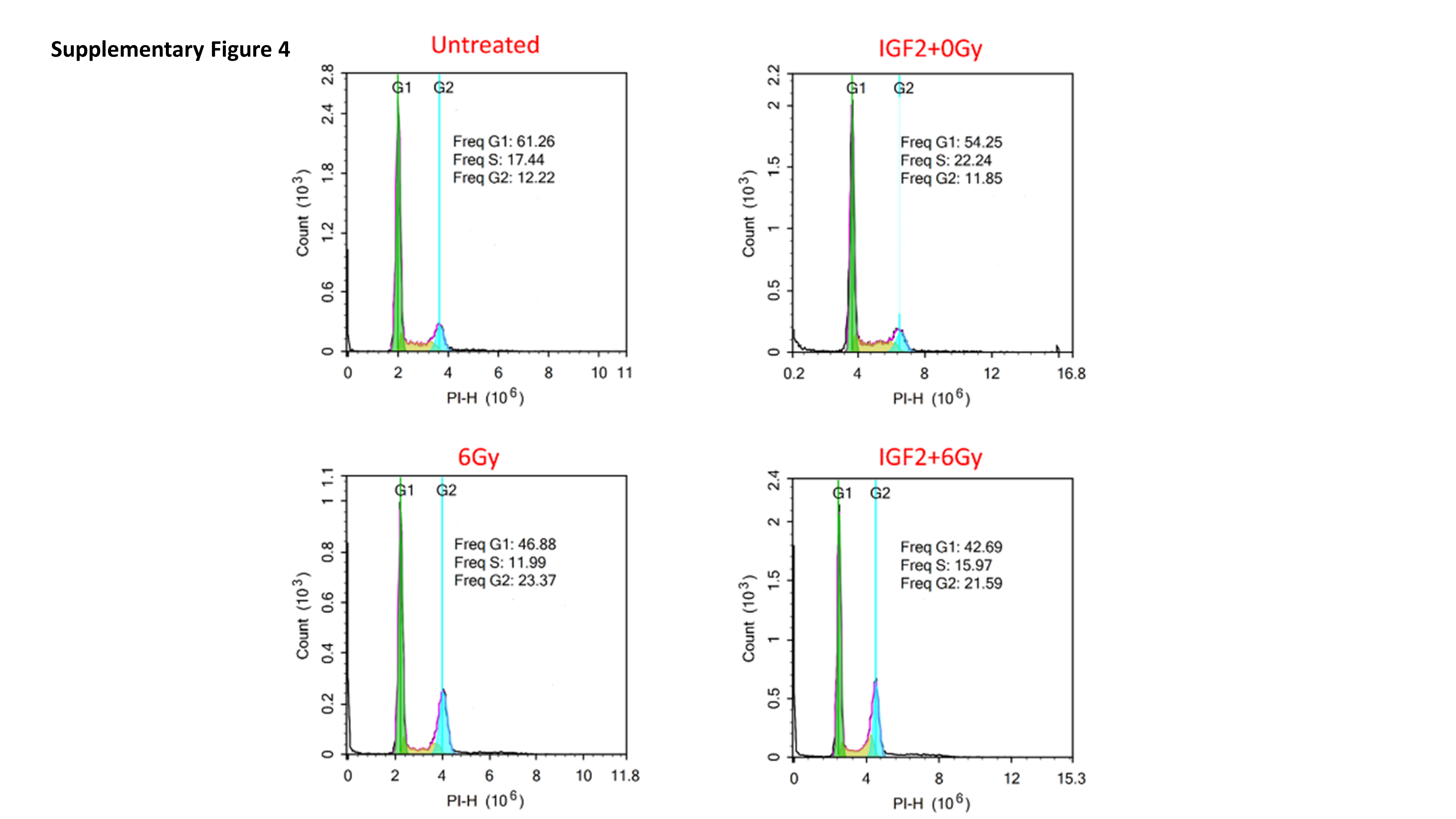

Supplement: Supplementary file 4 — Fig. S4. Flow cytometry of CADO cells showed no significant changes in cell cycle progression (amount of cells in G2/M phase) after IGF2 stimulation with or without irradiation (6 Gy). [file MOL2-14-1101-s004.tif]

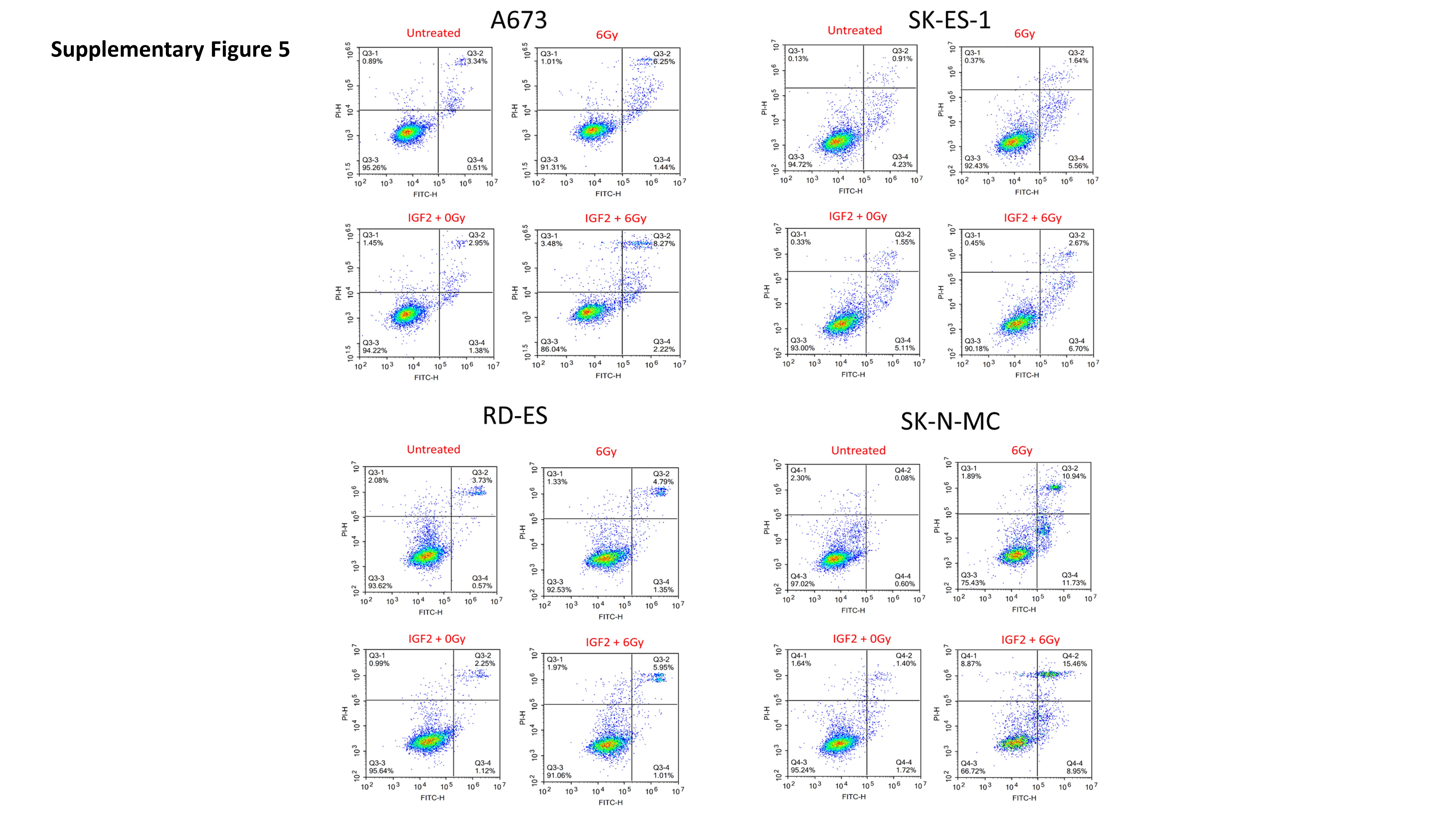

Supplement: Supplementary file 5 — Fig. S5. Flow cytometry was performed to determine the apoptotic rates of A673, SK‐ES‐1, RD‐ES and SK‐N‐MC ES cell lines. Two or more experiments were performed in each cell line. Quantification of the flow cytometry experiments are visualized as bar plots in Figure 3C. Upper right square = late apoptotic cells; Lower right square = early apoptotic cells; Upper right and lower right squares = total apoptotic cells. [file MOL2-14-1101-s005.tif]

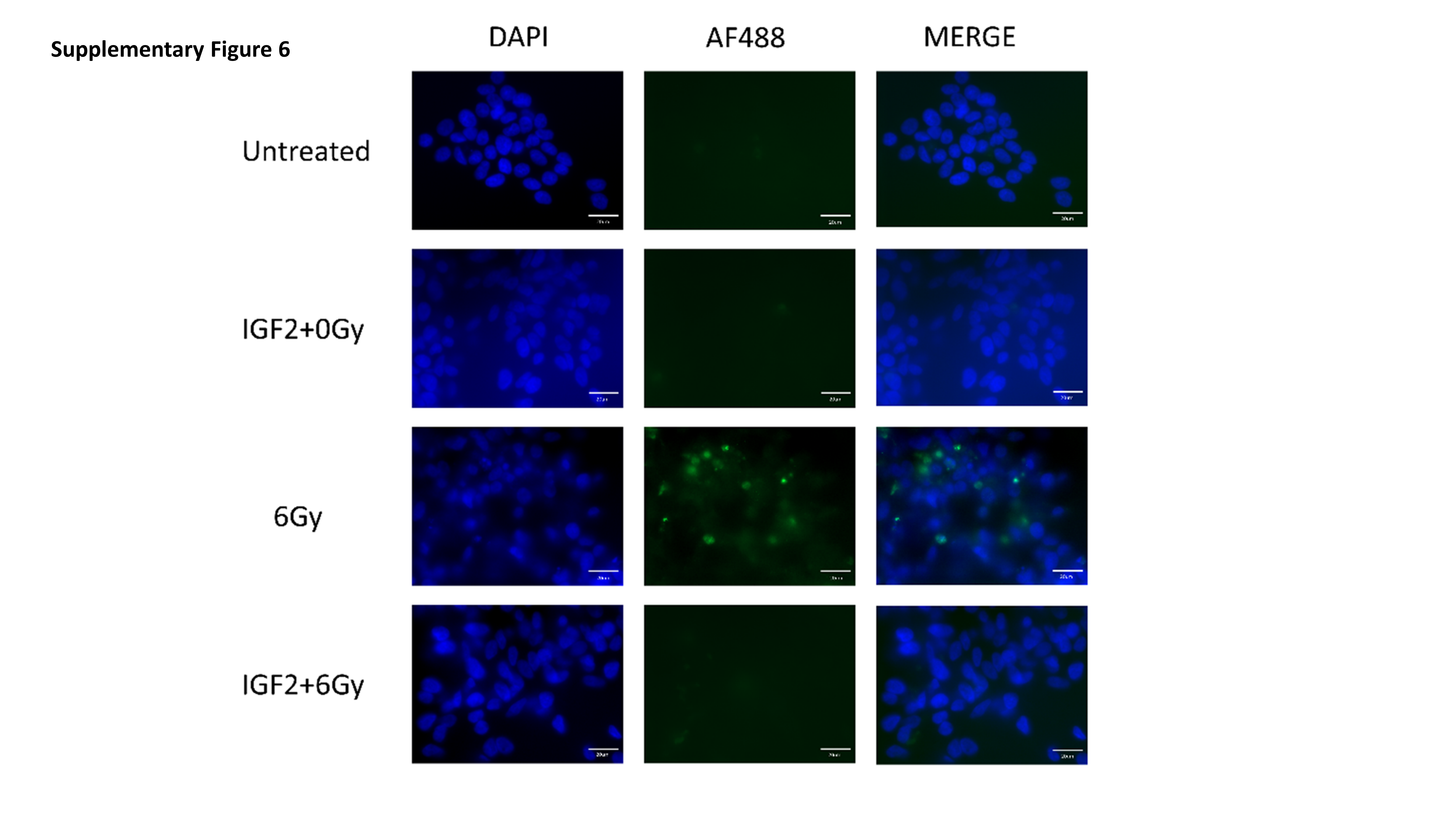

Supplement: Supplementary file 6 — Fig. S6. Immunofluorescence assays were conducted to detect how the levels of cleaved PARP was related to irradiation (6 Gy) with or without IGF2 stimulation in CADO cells. [file MOL2-14-1101-s006.tif]

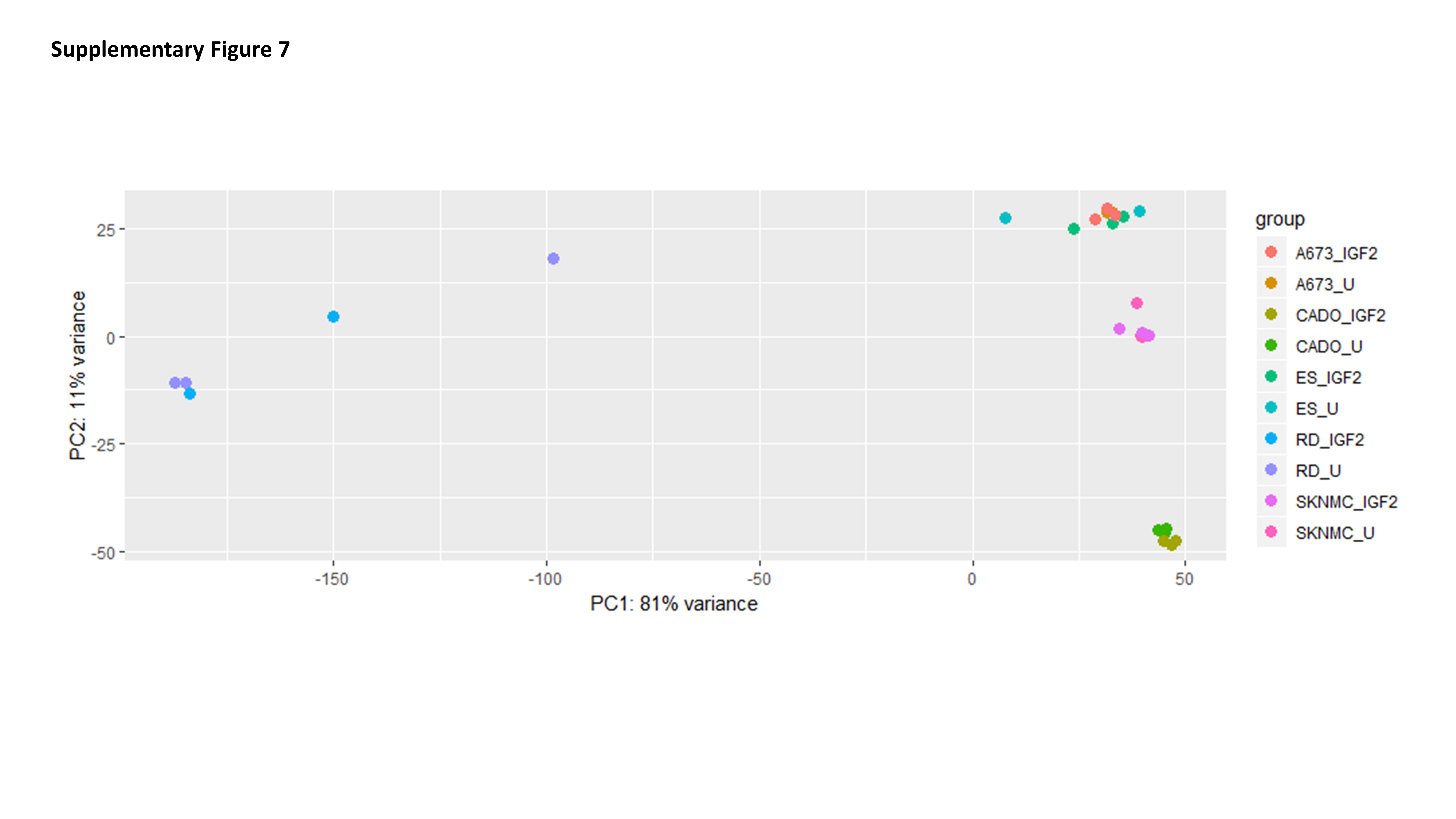

Supplement: Supplementary file 7 — Fig. S7. Principal component analysis (PCA) plot showing all genes in IGF2‐treated and untreated cell lines. Dots indicate individual replicates (n = 2 ‐ 3) and color represents cell line type and treatment (IGF2 = 100ng/ml IGF2 treatment, U = untreated) as indicated by the legend to the right. Poor separation was seen for RD‐ES cells which were excluded from further analysis. [file MOL2-14-1101-s007.tif]

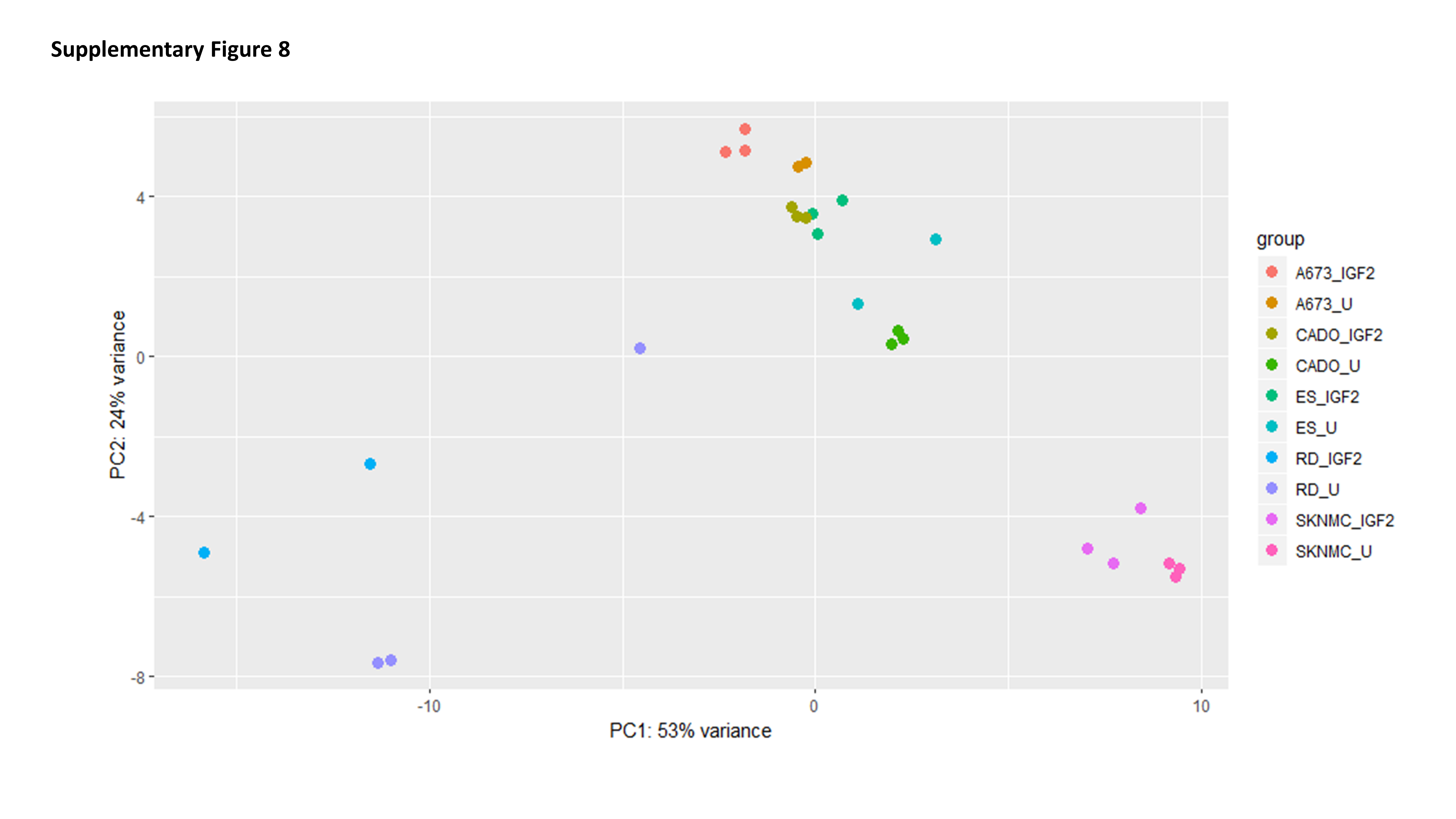

Supplement: Supplementary file 8 — Fig. S8. Principal component analysis (PCA) plot of all cell lines for the gene signature (36 differentially expressed genes) related to IGF2 stimulation of CADO cells. The PCA shows a variable separation (for each individual cell line) of treated and untreated experimental replicates. [file MOL2-14-1101-s008.tif]

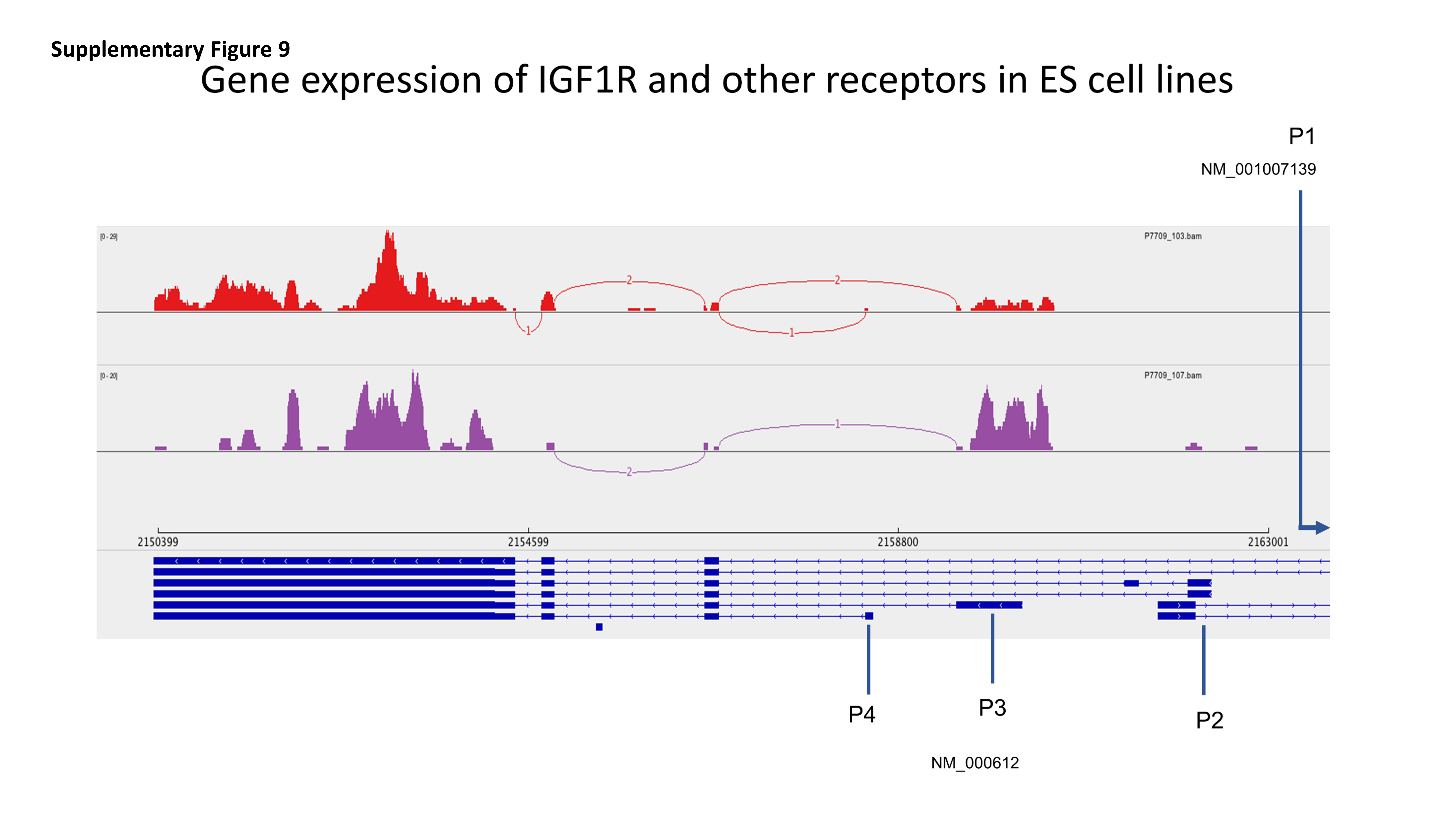

Supplement: Supplementary file 9 — Fig. S9. Sashimi plot showing the mapping of RNA‐seq reads to the IGF2 exons for a sample with IGF2 expression. Similar to the other cases with IGF2 expression on RNA‐seq, the mapping reveals how promoter P3 is the clearly dominant site for transcription initiation. Promoter P1 is not shown, but had a very low number of mapped reads for all samples. This suggests that the gene expression of IGF2 is not mediated by loss of imprinting. [file MOL2-14-1101-s009.tif]

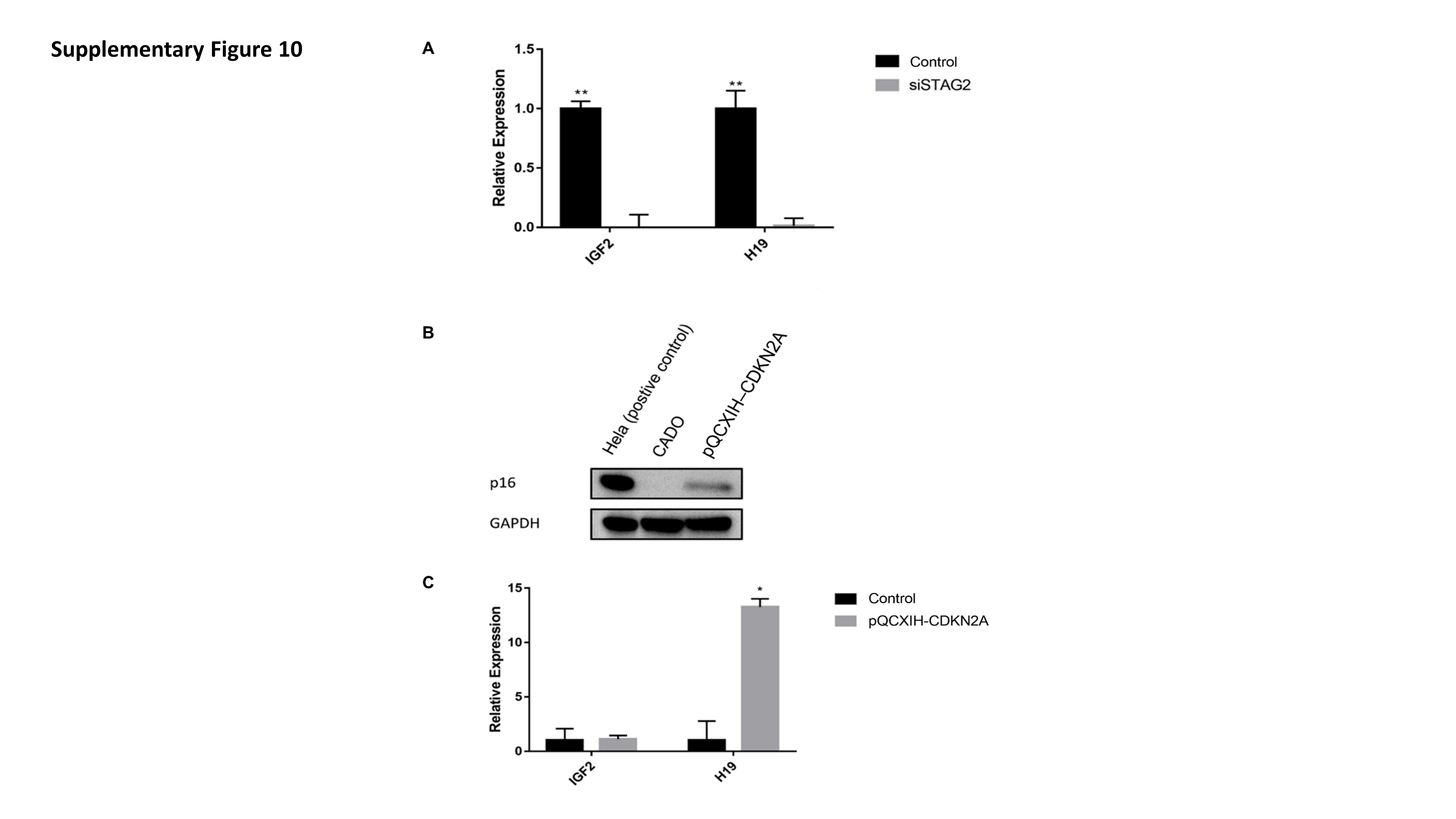

Supplement: Supplementary file 10 — Fig. S10. A) qRT–PCR analysis of IGF2 and H19 expression in untransfected control and siSTAG2‐treated CADO cells. B) Western blotting of P16 in Hela (positive control), uninfected CADO and CDKN2A encoding retrovirus infected (pQCXIH‐CDKN2A) CADO cells. GAPDH was stained as a loading control. C) qRT–PCR analysis of IGF2 and H19 expression in uninfected CADO and CDKN2A encoding retrovirus infected (pQCXIH‐CDKN2A) CADO cells. *p <0.05, **p <0.01. [file MOL2-14-1101-s010.tif]

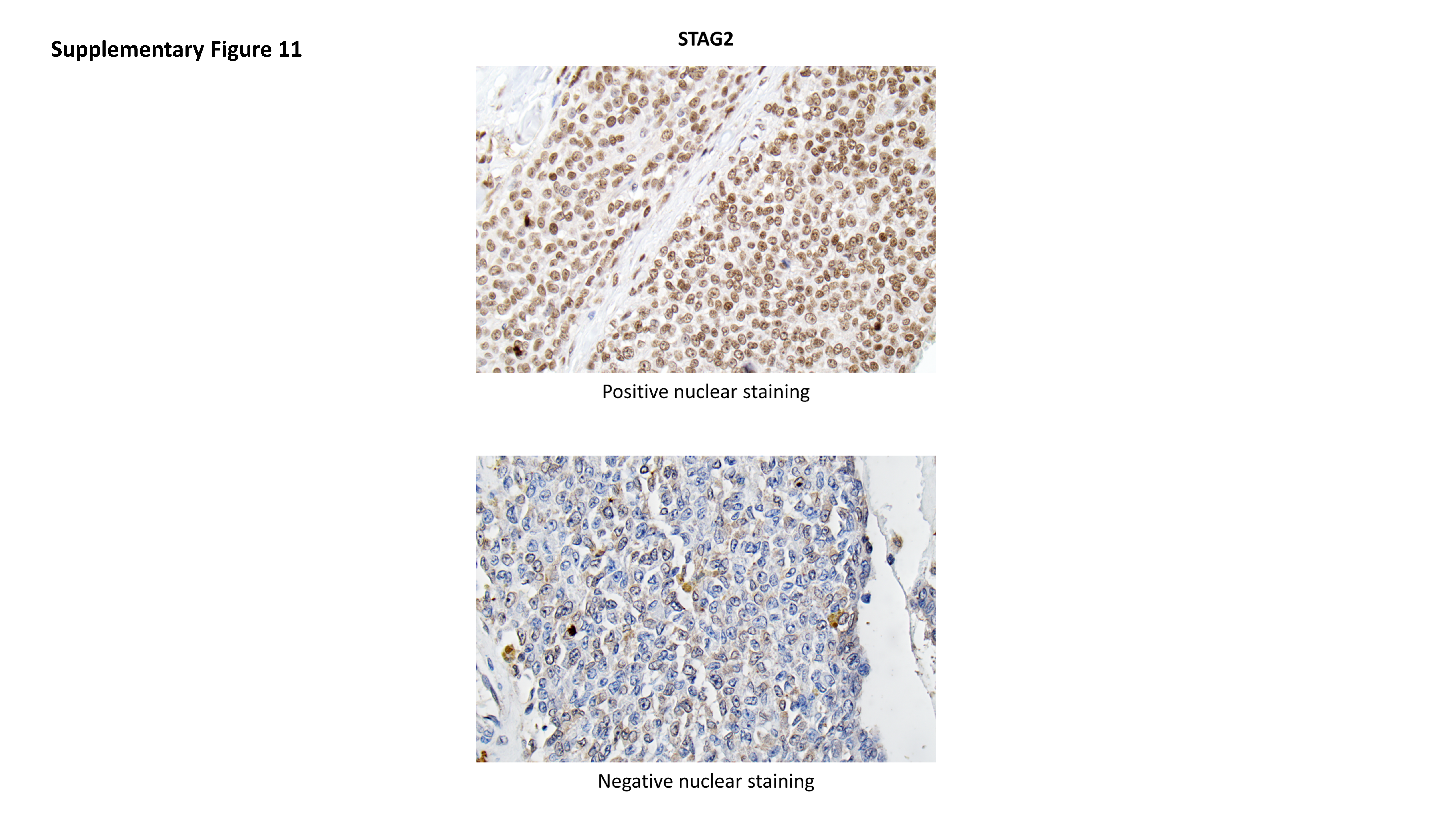

Supplement: Supplementary file 11 — Fig. S11. Representative photomicrographs of immunohistochemical staining for STAG2 in Ewing sarcomas. [file MOL2-14-1101-s011.tif]
